# Trends in Clinical Research Including Asian American, Native Hawaiian, and Pacific Islander Participants Funded by the US National Institutes of Health, 1992 to 2018

**DOI:** 10.1001/jamanetworkopen.2019.7432

**Published:** 2019-07-24

**Authors:** Lan N. Đoàn, Yumie Takata, Kari-Lyn K. Sakuma, Veronica L. Irvin

**Affiliations:** 1 College of Public Health and Human Sciences, School of Social and Behavioral Sciences, Oregon State University, Corvallis; 2 College of Public Health and Human Sciences, School of Biological and Population Health Sciences, Oregon State University, Corvallis

## Abstract

**Importance:**

Advancing the health equity agenda for Asian American, Native Hawaiian, and Pacific Islander (AA/NHPI) individuals has become an intersecting priority for federal agencies. However, the impact of federal investments and legislation to ensure systematic processes and resources to eliminate health disparities in AA/NHPI populations is unclear.

**Objective:**

To perform a portfolio review of clinical research funded by the National Institutes of Health (NIH) for AA/NHPI populations and determine the level of NIH investment in serving these populations.

**Design, Setting, and Participants:**

Cross-sectional study in which the NIH Research Portfolio Online Reporting Tools Expenditures and Results system was queried for extramural AA/NHPI-focused clinical research projects conducted in the United States from January 1, 1992, to December 31, 2018. Clinical research funded under research project grants, centers, cooperative awards, research career awards, training grants, and fellowships was included, with an advanced text search for AA/NHPI countries and cultures of origin. Project titles and terms were screened for inclusion and project abstracts were reviewed to verify eligibility. Descriptive analyses were completed.

**Main Outcomes and Measures:**

Outcomes included NIH funding trends and characteristics of funded projects and organizations. The proportions of AA/NHPI-related funding trends were calculated using 2 denominators, total NIH expenditures and clinical research expenditures.

**Results:**

There were 5460 records identified, of which 891 studies were reviewed for eligibility. Of these, 529 clinical research studies focused on AA/NHPI participants composed 0.17% of the total NIH budget over 26 years. Projects studying AA/NHPI individuals in addition to other populations were funded across 17 NIH institutes and centers. The top 5 funders collectively contributed almost 60% of the total funding dollars for AA/NHPI projects and were the National Cancer Institute ($231 584 664), National Institute on Aging ($108 365 124), National Heart, Lung, and Blood Institute ($67 232 910), National Institute on Minority Health and Health Disparities ($62 982 901), and National Institute on Mental Health ($60 072 779). Funding of these projects ($775 536 121) made up 0.17% of the overall NIH expenditures ($451 284 075 000) between 1992 and 2018, and 0.18% ($677 479 468) of the NIH research budget after 2000. Funding for AA/NHPI projects significantly increased over time, but the proportion of the total NIH budget has only increased from 0.12% before 2000 to 0.18% after 2000. Of total funding, 60.8% was awarded to research project grants compared with only 5.1% allocated to research career awards, training grants, and fellowships.

**Conclusions and Relevance:**

Increases in research dollars for AA/NHPI clinical research were not associated with increases in the overall NIH research budget, and underrepresentation of AA/NHPI subgroups still remains. Without overt direction from federal entities and dedicated funds for health disparities research, as well as parallel efforts to increase diversity in the biomedical workforce, investments may continue to languish for AA/NHPI populations.

## Key Points

**Question:**

What is the level of investment by the National Institutes of Health (NIH) to fund clinical research focused on Asian American, Native Hawaiian, and Pacific Islander populations?

**Findings:**

This cross-sectional study found 529 clinical research projects focused on Asian American, Native Hawaiian, and Pacific Islander participants funded by the NIH between 1992 and 2018, composing 0.17% of the total NIH budget. This proportion of the total NIH budget has only increased from 0.12% before 2000 to 0.18% after 2000.

**Meaning:**

These findings suggest that without overt direction from federal entities, dedicated funds for health disparities research, and parallel efforts to increase diversity in the biomedical workforce, investments may continue to languish for Asian American, Native Hawaiian, and Pacific Islander populations.

## Teaser

This cross-sectional study investigates spending by the US National Institutes of Health (NIH) for studies focusing on Asian American, Native Hawaiian, and Pacific Islander populations.

## Introduction

The landmark 1985 Report of the Secretary’s Task Force on Black and Minority Health (known as the Heckler Report)^1^ resulted in a national health focus on eliminating health disparities and concluded that Asian American, Native Hawaiian, and Pacific Islander (AA/NHPI) populations are healthier than all other racial/ethnic groups in the United States. The prevailing stereotype that AA/NHPI groups are model minority populations^2^ has resulted in data equity efforts negatively affecting AA/NHPI subgroups (eg, Vietnamese or Samoan).^3^ Data equity is the need for high-quality, disaggregated racial/ethnic data to capture disparities and underlying social factors associated with health needed to develop evidence-based solutions that inform public policies.

Asian American, Native Hawaiian, and Pacific Islander populations represent more than 50 countries or cultures of origin and 100 different languages and are the fastest-growing racial/ethnic group in the United States.^4,5^ Data for AA/NHPI populations are typically grouped together, which can conflate or inflate the magnitude of health and health outcomes. For example, in aggregate, AA/NHPI adult rates of liver cancer incidence and mortality are double those of non-Hispanic white adults.^6^ However, when data are disaggregated, liver cancer incidence is 7 times higher and 9 times higher for Laotian men and women, respectively, compared with non-Hispanic white adults.^6^ Understanding the complexities of health by subgroup could be the difference between eliminating or worsening health disparities.^7,8^

The health of AA/NHPI groups is further complicated when data are stratified by sociodemographic characteristics. In 2017, approximately half of AA/NHPI individuals were foreign born and recent immigrants entering the United States in 2000 or later.^9^ Asian American, Native Hawaiian, and Pacific Islander individuals were more likely to speak a language other than English compared with non-Hispanic white individuals.^9^ Despite the recognized heterogeneity across AA/NHPI subgroups, these populations remain understudied,^7,10,11^ and data collection, reporting, and dissemination issues^4,12,13,14^ challenge the ability to understand health.^15^ Most federal databases are limited to simple distributions owing to small sample sizes for AA/NHPI groups.^16^ Furthermore, inconsistent implementation of racial/ethnic classifications and dearth of culturally appropriate instruments decrease our ability to understand exposures and health outcomes across subgroups.^4^

Between 1986 and 2000, Ghosh^11^ found that AA/NHPI participants were represented in 0.2% of all health-related grants from 7 federal agencies. Similarly, an average investment of 0.4% in AA/NHPI communities was found in the top 20 major US foundations.^17^ Taken together, there have been minimal financial investments in AA/NHPI populations by federal agencies and philanthropy, even though AA/NHPI individuals represent more than 5.0% of the total US population.^9^

During the past 2 decades, notable efforts by the federal government have emphasized reducing health disparities for AA/NHPI individuals.^4,14^ The National Institutes of Health (NIH) Revitalization Act of 1993^18,19^ and Minority Health and Health Disparities Research and Education Act of 2000^20^ established the National Institute on Minority Health and Health Disparities. Likewise, the NIH Health Disparities Strategic Plan and Budget^21^ prioritized eliminating health disparities in racial/ethnic minority populations. The 1997 Office of Management and Budget Directive 15^22^ recognized AA/NHPI as 2 separate racial categories, and the Patient Protection and Affordable Care Act section 4302 expanded these data collection standards to include 7 AA and 4 NHPI subgroups.^23,24^ In 2009, Executive Order 13515^25^ reestablished the Office of the White House Initiative on Asian Americans and Pacific Islanders that highlighted more AA/NHPI-focused investigations.^26^

Advancing an inclusive national agenda for AA/NHPI populations has become an intersecting priority for federal agencies.^7,8^ However, the impact of federal investments and legislation to ensure systematic processes and resources to eliminate health disparities in AA/NHPI groups is unclear. The purpose of this portfolio review is to examine the level of investment by the NIH that focused on clinical research in AA/NHPI populations.

## Methods

The NIH Research Portfolio Online Reporting Tools Expenditures and Results (RePORTER) system^27^ is an electronic database of federally funded projects dating from 1985 to the present and includes project information and abstracts. We focused on NIH grant programs because the agency is the largest funder of health research globally.^28^ Approval of this study was waived by the Oregon State University institutional review board because it did not involve human participants.

### Project Searching

We used NIH RePORTER to collect information on extramural NIH-funded clinical research projects conducted in the US and associated territories and funded between January 1, 1992, and December 31, 2018. The NIH defines clinical research as patient-oriented research, epidemiologic and behavioral studies, and outcomes and health services research.^29^ We defined unique projects as having the same grant number, which may include multiple years of funding. We excluded active projects because they have not completed the budget period or are projects with no-cost extensions. We included research project grants, centers, cooperative agreements, research career awards, training grants, and fellowships and performed an advanced text search for key words representing AA/NHPI countries or cultures of origin (eMethods in the Supplement).

### Data Extraction and Qualitative Synthesis

We exported projects matching the search criteria, screened project titles and terms for inclusion, and reviewed project abstracts for eligibility. We classified projects into 2 categories:

1. AA/NHPI only, which includes only AA/NHPI participants, as grouped or disaggregated ethnicities (eg, project included Vietnamese and Chinese participants or Asian participants);
2. AA/NHPI plus non-AA/NHPI, which includes AA/NHPI participants and additional racial/ethnic groups (eg, project included Asian, black or African American, and non-Hispanic white participants).

Total AA/NHPI is the sum of the 2 categories. We excluded projects that did not fit the clinical research definition, did not explicitly include AA/NHPI participants, took place internationally, or had no project abstracts.

We cataloged studies under specific criteria, including the primary target populations, AA/NHPI subgroups, health domain, and geographic region. Project health domains were coded using the NIH Research, Condition, and Disease Categorization (RCDC) system,^30^ which uses text data mining and NIH-defined terms to catalog projects into research areas. We coded whether projects were trans-NIH efforts (eg, funded by >1 of the NIH Institutes or Centers [ICs]) or funded under multiple funding opportunity announcements (FOAs). The first author (L.N.Đ.) identified the clinical research studies based on search criteria that were discussed with the last author (V.L.I.). We followed the Preferred Reporting Items for Systematic Reviews and Meta-analyses (PRISMA) protocol^31^ for the search strategy, study selection, and data extraction from project abstracts. The last author (V.L.I.) was consulted for study eligibility and categorization when necessary. The first author (L.N.Đ.) coded whether the FOAs specifically referenced AA/NHPI participants or health disparities populations. Geographic regions were based on NIH RePORTER^32^ regional grouping and US Census Bureau definitions.

### Statistical Analysis

Descriptive analyses included NIH dollar investments over time, characteristics of AA/NHPI clinical research, and features of the funded organizations. We examined the proportions of AA/NHPI-related research using 2 denominators, total NIH expenditures and clinical research expenditures. We calculated the annual changes in funding amounts and new projects awarded between 1992 and 2018 using simple linear regression. We also compared funding amounts and projects awarded before 2000 and after 2000, to compare with the study period used by Ghosh.^11^ We defined statistical significance as *P* < .05. Data were managed in Microsoft Excel (Microsoft Corp) and we conducted statistical analysis using R statistical software version 3.5.0 (R Project for Statistical Computing).

We obtained the total NIH expenditures from the NIH Office of Budget Mechanism Detail for Total NIH for fiscal years 1992 to 2018.^33,34^ Total NIH expenditures were defined as total research grants (not including small business innovation research/small business technology transfer grants) plus total research training. Clinical research expenditures for fiscal years 2008 to 2018 were obtained from the Estimates for Funding for Various RCDC.^30^

## Results

**Figure 1.**
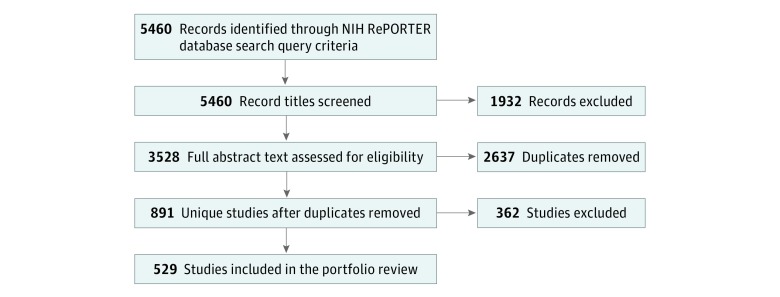
PRISMA Flow Diagram for Inclusion of Projects Into Portfolio Review Project titles and abstracts were screened for relevance and eligibility. Duplicates were removed based on unique serial numbers. The fiscal year total costs were combined to show fiscal year total costs by year for each unique grant. Reasons for exclusion included the following: did not reference inclusion of Asian American, Native Hawaiian, and Pacific Islander participants in research; did not take place in the United States; no abstract available; and did not fit the definition of clinical research (eg, animal, genetic, and cellular research). The portfolio analysis included summarizing longitudinal funding trends for clinical research focused on Asian American, Native Hawaiian, and Pacific Islander participants funded by the National Institutes of Health and descriptive information of included population subgroups, administering Institutes and Centers, funding mechanisms, project health domains, and funded organization characteristics. RePORTER indicates Research Portfolio Online Reporting Tools Expenditures and Results.

We identified 5460 records on NIH RePORTER based on our search criteria (Figure 1). After screening project titles and project terms, we removed 2637 duplicate records and reviewed 891 project abstracts for inclusion. We included 529 unique projects in this analysis.

### Institutes and Centers

Total AA/NHPI projects were funded across 17 NIH ICs (Table 1). The top 5 funders collectively contributed almost 60% of the total funding dollars for AA/NHPI projects and were the National Cancer Institute ($231 584 664), National Institute on Aging ($108 365 124), National Heart, Lung, and Blood Institute ($67 232 910), National Institute on Minority Health and Health Disparities ($62 982 901), and National Institute on Mental Health ($60 072 779). The ICs awarding the greatest number of projects were the National Cancer Institute (n = 132), the National Institute on Mental Health (n = 62), the National Institute on Aging (n = 57), the National Institute of Child Health and Human Development (n = 48), and the National Institute of Nursing Research (n = 37). By funding mechanisms, the National Cancer Institute awarded the most research project grants (n = 102) and cooperative agreements (n = 14), the National Center for Research Resources awarded the most centers (n = 15), the National Institute on Mental Health awarded the most research career awards (n = 14), the National Institute of General Medical Sciences awarded the most training grants (n = 6), and the National Institute of Nursing Research awarded the most fellowships (n = 15).

**Table 1.** Administering National Institutes of Health Institutes and Centers

Institutes and Centers	Awards, No. (%)	Total Funding Amount, $	Funding per Project, Mean (SD), $^a^
National Cancer Institute	132 (25.0)	231 584 664	1 754 429 (3 031 995)
National Institute on Mental Health	62 (11.7)	60 072 779	968 916 (1 167 984)
National Institute on Aging	57 (10.8)	108 365 124	1 901 143 (3 637 932)
Eunice Kennedy Shriver National Institute of Child Health and Human Development	48 (9.1)	48 992 219	1 020 671 (1 426 028)
National Institute of Nursing Research	37 (7.0)	22 691 061	613 272 (672 124)
National Institute on Minority Health and Health Disparities	34 (6.4)	62 982 901	1 852 438 (2 235 275)
National Institute on Drug Abuse	33 (6.2)	37 276 683	1 129 596 (1 154 949)
National Heart, Lung, and Blood Institute	31 (5.9)	67 232 910	2 168 804 (1 958 352)
National Institute of Diabetes and Digestive and Kidney Diseases	26 (4.9)	31 638 678	1 216 872 (1 013 336)
National Institute on Alcohol Abuse and Alcoholism	18 (3.4)	16 091 232	893 957 (964 628)
National Center for Research Resources	17 (3.2)	30 236 345	1 778 609 (4 722 591)
National Center for Complementary and Integrative Health	9 (1.7)	2 596 203	288 467 (150 852)
National Institute of General Medical Sciences	8 (1.5)	17 389 974	2 173 747 (1 864 988)
National Institute of Environmental Health Sciences	6 (1.1)	8 203 552	1 367 259 (1 004 345)
National Institute of Dental and Craniofacial Research	6 (1.1)	10 977 711	1 829 619 (2 392 057)
National Institute of Arthritis and Musculoskeletal and Skin Diseases	3 (0.6)	3 649 093	1 216 364 (498 276)
National Institute of Neurological Disorders and Stroke	2 (0.4)	15 554 992	7 777 496 (9 180 988)

^a^ Mean funding amount was calculated by total funding amount divided by the number of grants.

Total AA/NHPI projects were funded under 262 unique FOAs, in which 49 projects (9.3%) were trans-NIH efforts and 17 projects (3.2%) were funded under more than 1 FOA. Approximately 22.5% of the FOAs explicitly referenced AA/NHPI populations under the funding opportunity description and 29.4% of the FOAs referenced health disparities population terms (eTable 1 in the Supplement).

### Funding Mechanisms

Table 2 shows the allocation of NIH dollar amounts by funding mechanisms and grouping category. The NIH funded a total of $775 536 121 toward 529 research projects, with 60.8% of total AA/NHPI funding allocated to research project grants ($471 406 495), 18.3% to centers ($141 828 462), 15.8% to cooperative agreements ($122 809 956), and less than 5.1% to research career awards ($24 458 837), training grants ($10 821 202), and fellowships ($4 211 169). More than two-thirds of the total AA/NHPI projects awarded were research project grants (n = 360), followed by centers (n = 57), research career awards (n = 41), fellowships (n = 39), cooperative agreements (n = 25), and training grants (n = 7).

**Table 2.** Dollar Amounts Allocated to Clinical Research Grants by Funding Mechanism by Grouping Category

Funding Mechanism	AA/NHPI Only			AA/NHPI and Non-AA/NHPI			Total AA/NHPI		
	Awards, No. (%)	Total Funding Amount, $	Funding per Project, Mean (SD), $^a^	Awards, No. (%)	Total Funding Amount, $	Funding per Project, Mean (SD), $^a^	Awards, No. (%)	Total Funding Amount, $	Funding per Project, Mean (SD), $^a^
Total	262	409 297 167	1 562 203 (2 635 700)	267	366 238 954	1 371 681 (2 275 691)	529	775 536 121	1 466 042 (2 462 430)
Research project grants^b^	173 (66.0)	234 319 617	1 354 449 (1 522 477)	187 (70.0)	237 086 878	1 267 844 (1 903 319)	360 (68.1)	471 406 495	1 309 462 (1 728 966)
R01	98 (37.4)	214 141 949	2 185 122 (1 574 276)	125 (46.8)	217 973 811	1 743 790 (2 146 826)	223 (42.2)	432 115 760	1 937 739 (1 924 941)
R03	33 (12.6)	3 668 536	111 168 (50 191)	31 (11.6)	3 982 107	128 455 (57 584)	64 (12.1)	7 650 643	119 541 (54 170)
R21	40 (15.3)	15 408 755	385 219 (134 198)	26 (9.7)	8 826 097	339 465 (133 316)	66 (12.5)	24 234 852	367 195 (134 717)
R29	2 (0.8)	1 100 377	550 189 (29 603)	3 (1.1)	1 228 015	409 338 (6696)	5 (0.9)	2 328 392	465 678 (78 696)
RF1	0	0	0	2 (0.7)	5 076 848	2 538 424 (2 314 314)	2 (0.4)	5 076 848	2 538 424 (2 314 314)
Centers^c^	20 (7.6)	67 996 699	3 399 835 (5 784 487)	37 (13.9)	73 831 763	1 995 453 (3 110 237)	57 (10.8)	141 828 462	2 488 219 (4 246 005)
M01	9 (3.4)	1 248 682	138 742 (105 609)	4 (1.5)	802 093	200 523 (158 436)	13 (2.5)	2 050 775	157 752 (120 797)
P01	3 (1.1)	42 769 892	14 256 631 (7 083 107)	6 (2.2)	14 168 673	2 361 446 (2 232 202)	9 (1.7)	56 938 565	6 326 507 (7 143 575)
P20	3 (1.1)	14 058 919	4 686 306 (4 862 502)	10 (3.7)	25 818 539	2 581 854 (3 584 057)	13 (2.5)	39 877 458	3 067 497 (3 798 214)
P30	3 (1.1)	2 249 079	749 693 (530 793)	9 (3.4)	9 825 320	1 091 702 (1 292 336)	12 (2.3)	12 074 399	1 006 200 (1 135 691)
P50	2 (0.8)	7 670 127	3 835 064 (3 424 621)	8 (3.0)	23 217 138	2 902 142 (4 828 966)	10 (1.9)	30 887 265	3 088 727 (4 426 598)
Cooperative agreements^d^	12 (4.6)	85 317 706	7 109 809 (4 801 800)	13 (4.9)	37 492 250	2 884 019 (4 833 542)	25 (4.7)	122 809 956	4 912 398 (5 185 787)
U54	12 (4.6)	85 317 706	7 109 809 (4 801 800)	13 (4.9)	37 492 250	2 884 019 (4 833 542)	25 (4.7)	122 809 956	4 912 398 (5 185 787)
Research career awards^e^	29 (11.1)	17 218 461	593 740 (246 272)	12 (4.5)	7 240 376	603 365 (244 844)	41 (7.8)	24 458 837	596 557 (242 817)
K01	12 (4.6)	7 291 615	607 635	5 (1.9)	2 348 037	469 607 (267 855)	17 (3.2)	9 639 652	567 038 (250 550)
K02	2 (0.8)	1 083 896	541 948 (140 816)	0	0	0	2 (0.4)	1 083 896	541 948 (140 816)
K07	4 (1.5)	1 540 581	385 145 (270 797)	0	0	0	4 (0.8)	1 540 581	385 145 (270 797)
K08	2 (0.8)	1 322 543	661 272 (326 413)	1 (0.4)	581 682	581 682	3 (0.6)	1 904 225	634 742 (235 338)
K23	9 (3.4)	5 979 826	664 425 (241 306)	3 (1.1)	1 937 425	645 808 (71 552)	12 (2.3)	7 917 251	659 771 (208 206)
K24	0	0	0	3 (1.1)	2 373 232	791 077 (283 877)	3 (0.6)	2 373 232	791 077 (283 877)
Training grants^f^	1 (0.4)	1 329 824	1 329 824	6 (2.2)	9 491 378	1 581 896 (1 136 902)	7 (1.3)	10 821 202	1 545 886 (1 042 209)
T32	0	0	0	1 (0.4)	930 450	930 450	1 (0.2)	930 450	930 450
T34	1 (0.4)	1 329 824	1 329 824	5 (1.9)	8 560 928	1 712 186 (1 219 987)	6 (1.1)	9 890 752	1 648 459 (1 102 298)
Fellowships^g^	27 (10.3)	3 114 860	115 365 (293 741)	12 (4.5)	1 096 309	91 359 (51 979)	39 (7.4)	4 211 169	107 979 (244 836)
F31	26 (9.9)	3 067 076	117 964 (299 242)	9 (3.4)	644 467	71 607 (44 130)	35 (6.6)	3 711 543	106 044 (258 308)
F32	1 (0.4)	47 784	47 784	3 (1.1)	451 842	150 614 (6914)	4 (0.8)	499 626	124 907 (51 724)

Abbreviation: AA/NHPI, Asian American, Native Hawaiian, and Pacific Islander.

^a^ Mean funding amount was calculated by total funding amount divided by the number of grants.

^b^ Research project grants included R01, Research Project; R03, Small Research Grants; R21, Exploratory/Developmental Grants; R29, First Independent Research Support & Transition Awards; and RF1, Multi-Year Funded Research Project Grant.

^c^ Centers included M01, General Clinical Research Centers Program; P01, Research Program Projects; P20, Exploratory Grants; and P50, Specialized Center.

^d^ Cooperative Agreements included U54, Specialized Centers.

^e^ Research career awards included K01, Research Scientist Development Award—Research & Training; K02, Research Scientist Development Award—Research; K07, Academic/Teacher Award; K08, Clinical Investigator Award; K23, Mentored Patient-Oriented Research Career Development Award; and K24, Midcareer Investigator Award in Patient-Oriented Research.

^f^ Training grants included the T32, Institutional National Research Service Award and T34, Undergraduate National Research Service Award Institutional Research Training Grants.

^g^ Fellowships included the F31, Predoctoral Individual National Research Service Award and F32, Postdoctoral Individual National Research Service Award.

Amounts of AA/NHPI-only funding were greater for overall research project grants, cooperative agreements, and fellowships but lower for center, career, and training grants compared with AA/NHPI plus non-AA/NHPI funding amounts. Projects that were AA/NHPI only received less in total funding dollars ($234 319 617) but had a greater mean funding per project ($1 354 449) compared with AA/NHPI plus non-AA/NHPI projects. Looking at R01 grants, the AA/NHPI-only R01 budget ($214 141 949) was less than the AA/NHPI plus non-AA/NHPI R01 budget ($217 973 811), but the mean per project was more for AA/NHPI-only R01s ($2 185 122). Mean project funding was greater for AA/NHPI only for research project grants ($1 574 372), centers ($3 399 835), and fellowships ($115 365) compared with AA/NHPI plus non-AA/NHPI research project grants ($1 267 844), centers ($1 995 453), and fellowships ($91 359).

### Project Health Domains and Funded Organizations

Table 3 shows the project health domains and funded organizations type and regional distribution. The most common health domains were cancer (n = 116), mental health (n = 74), cardiometabolic disease (n = 51), cardiovascular disease (n = 30), and substance abuse (n = 24).

**Table 3.** Project and Organizational Characteristics of Clinical Research Included in the Portfolio Review

Project and Organization Characteristic	Awards, No. (%)	Total Funding Amount, $	Funding per Project, Mean (SD), $^a^
**Project Health Domains (n = 29)**^**b**^			
Cancer	116 (21.9)	189 202 228	1 631 054 (2 922 559)
Mental health	74 (14.0)	93 262 509	1 260 304 (1 951 206)
Cardiometabolic diseases	51 (9.6)	84 668 549	1 660 168 (2 831 986)
Cardiovascular disease	30 I5.7)	74 681 214	2 489 374 (3 303 152)
Substance abuse	24 (4.5)	19 779 521	824 147 (1 031 652)
Health disparities	22 (4.2)	53 030 278	2 410 467 (3 546 635)
Alcoholism, alcohol use, and health	21 (4.0)	17 887 555	851 788 (1 032 028)
Aging	20 (3.8)	12 710 684	635 534 (817 671)
HIV/AIDS	20 (3.8)	29 936 151	1 496 808 (1 005 935)
Maternal and child health	18 (3.4)	13 425 811	745 878 (632 907)
Capacity building	16 (3.0)	21 997 429	1 374 839 (1 822 450)
Smoking and health	15 (2.8)	18 583 523	1 238 902 (1 073 066)
Alzheimer disease related dementias	13 (2.5)	54 716 013	4 208 924 (6 346 156)
Complementary and alternative medicine	10 (1.9)	4 485 345	448 535 (418 146)
Health services	10 (1.9)	8 812 266	881 227 (578 371)
Research training	8 (1.5)	11 255 232	1 406 904 (1 038 561)
Osteoporosis	7 (1.3)	4 994 537	713 505 (601 676)
Environmental exposure	7 (1.3)	8 611 067	1 230 152 (985 991)
Women's health	7 (1.3)	9 256 400	1 322 343 (1 215 306)
Child development	6 (1.1)	2 255 945	375 991 (263 339)
Sleep research	6 (1.1)	9 323 543	1 553 924 (1 245 418)
Methods	5 (0.9)	2 166 769	433 354 (349 972)
Oral health	5 (0.9)	10 488 590	2 097 718 (2 571 642)
Migration	3 (0.6)	1 177 560	392 520 (413 595)
Physical activity	3 (0.6)	2 091 441	697 147 (862 439)
Organ transplantation	3 (0.6)	2 432 256	810 752 (325 625)
Caregiving research	3 (0.6)	2 546 885	848 962 (991 357)
Suicide	3 (0.6)	3 278 304	1 092 768 (977 193)
Other	3 (0.6)	8 478 516	2 826 172 (2 898 775)
**Organization Type (n = 16)**^**c**^			
Schools of medicine	128 (24.2)	182 857 757	1 428 576 (2 280 052)
Research institutes	73 (13.8)	110 419 596	1 512 597 (1 829 595)
Schools of arts and sciences	64 (12.1)	51 397 258	803 082 (962 098)
Domestic higher education	59 (11.2)	187 192 280	3 172 751 (4 569 862)
Schools of nursing	48 (9.1)	39 081 199	814 192 (827 794)
Schools of public health	44 (8.3)	47 467 675	1 078 811 (1 155 727)
Organized research units	27 (5.1)	57 352 298	2 124 159 (4 408 014)
Independent hospitals	19 (3.6)	24 532 390	1 291 178 (1 843 050)
Other domestic higher education	17 (3.2)	19 379 786	1 139 987 (1 513 222)
Schools of social work	17 (3.2)	14 845 441	873 261 (881 877)
Other domestic nonprofits	14 (2.6)	21 745 731	1 553 267 (2 141 595)
Schools of allied health professions	9 (1.7)	11 899 652	1 322 184 (1 087 483)
Schools of education	6 (1.1)	4 049 971	674 995 (1 099 251)
Schools of dentistry	2 (0.4)	2 951 074	1 475 537 (1 381 426)
Schools of pharmacy	1 (0.2)	205 013	205 013
Schools of earth sciences and natural resources	1 (0.2)	159 000	159 000
**Project and Organization Characteristics**			
Geographic region and states^d^			
Western	267 (50.5)	492 415 443	1 844 253 (3 051 301)
California	170 (32.1)	316 509 458	1 861 820 (3 042 723)
Hawaii	47 (8.9)	108 663 810	2 311 996 (3 807 866)
Washington	35 (6.6)	36 306 922	1 037 341 (1 154 741)
Oregon	6 (1.1)	15 149 379	2 524 897 (2 563 994)
Arizona	6 (1.1)	13 898 016	2 316 336 (4 994 363)
Colorado	2 (0.4)	1 032 035	516 018 (531 833)
New Mexico	1 (0.2)	855 823	855 823
Eastern	107 (20.2)	128 958 357	1 205 218 (1 683 170)
New York	44 (8.3)	41 251 216	937 528 (1 288 963)
Massachusetts	26 (4.9)	34 881 874	1 341 611 (1 879 742)
Pennsylvania	25 (4.7)	46 047 153	1 841 886 (2 202 227)
Connecticut	4 (0.8)	3 161 657	790 414 (1 120 691)
New Jersey	4 (0.8)	2 140 843	535 211 (579 052)
Rhode Island	3 (0.6)	800 185	266 728 (300 984)
Delaware	1 (0.2)	675 429	675 429
Central	83 (15.7)	68 356 646	823 574 (985 525)
Illinois	35 (6.6)	33 703 581	962 959 (978 201)
Minnesota	15 (2.8)	12 808 466	853 898 (1 003 358)
Michigan	11 (2.1)	6 318 674	574 425 (632 666)
Wisconsin	7 (1.3)	8 874 550	1 267 793 (1 817 288)
Indiana	4 (0.8)	1 731 998	433 000 (741 282)
Iowa	3 (0.6)	880 191	293 397 (217 281)
Missouri	3 (0.6)	890 071	296 690 (220 898)
Ohio	2 (0.4)	827 616	413 808 (367 601)
Kansas	1 (0.2)	1 864 276	1 864 276
Nebraska	1 (0.2)	387 748	387 748
North Dakota	1 (0.2)	69 475	69 475
Southern	71 (13.4)	72 313 994	1 018 507 (1 267 911)
Maryland	23 (4.3)	20 878 702	907 770 (883 458)
Texas	13 (2.5)	7 568 518	582 194 (556 600)
District of Columbia	11 (2.1)	13 431 754	1 221 069 (1 201 689)
North Carolina	7 (1.3)	7 793 672	1 113 382 (662 161)
Florida	5 (0.9)	1 493 700	298 740 (338 713)
Louisiana	3 (0.6)	7 289 105	2 429 702 (3 729 093)
Georgia	2 (0.4)	407 860	203 930 (238 982)
Tennessee	2 (0.4)	8 989 061	4 494 531 (2 359 688)
Alabama	1 (0.2)	2 355 990	2 355 990
Kentucky	1 (0.2)	1 146 540	1 146 540
Arkansas	1 (0.2)	425 292	425 292
Mississippi	1 (0.2)	440 308	440 308
Virginia	1 (0.2)	93 492	93 492
Territories	1 (0.2)	13 491 681	13 491 681
Guam	1 (0.2)	13 491 681	13 491 681

^a^ Mean funding amount was calculated by total funding amount divided by the number of grants.

^b^ The National Institutes of Health Research, Condition, and Disease Categorization system^30^ was used as a reference for the project health domains.

^c^ Organization types are based on National Institutes of Health Research Portfolio Online Reporting Tools Expenditures and Results^27^ classifications, defined by the function, mission, or service of the organization receiving a grant, contract, or cooperative agreement.

^d^ Geographic regions are based on National Institutes of Health Research Portfolio Online Reporting Tools Expenditures and Results^32^ regional grouping and US Census Bureau definitions.

There were 161 unique grantee organizations funded. More than 76% of total AA/NHPI funding was concentrated in 5 organization types, including domestic higher education ($187 192 280), schools of medicine ($182 857 757), research institutes ($110 419 596), organized research units ($57 352 298), and schools of arts and sciences ($51 397 258). The greatest number of projects were awarded to schools of medicine (n = 128), followed by research institutes (n = 73), schools of arts and sciences (n = 64), domestic higher education (n = 59), and schools of nursing (n = 48).

By geographic region, 63.5% of the total AA/NHPI funding amount ($492 415 443) and more than half of the total AA/NHPI projects (n = 267) were awarded to organizations located in the Western region, followed by 16.6% of the total AA/NHPI funding ($128 958 357) awarded to 107 projects in the Eastern region. More than 62.6% of the total AA/NHPI projects were conducted in 5 states, California (n = 170), Hawaii (n = 47), New York (n = 44), Washington (n = 35), and Illinois (n = 35), with more than 50% allocated to California ($316 509 458).

### Disaggregated Race/Ethnicity

Almost 60% of the total AA/NHPI projects (n = 303) mentioned an AA/NHPI subgroup (eg, the project abstract explicitly mentioned Vietnamese participants). More than 75.0% of AA/NHPI-only projects (n = 200) specified an AA/NHPI subgroup compared with 38.0% of AA/NHPI plus non-AA/NHPI projects (n = 103). Among AA/NHPI-only projects, the subgroups most represented were Chinese (n = 71), Korean (n = 42), Vietnamese (n = 37), Filipino (n = 21), and Japanese (n = 19) for AA participants, and Native Hawaiian (n = 18), Samoan (n = 7), Marshallese (n = 4), Chamorro (n = 3), and Tongan (n = 3) for NHPI participants. For AA/NHPI plus non-AA/NHPI projects, Chinese (n = 62), Filipino (n = 20), Japanese (n = 18), Native Hawaiian (n = 15), and Vietnamese (n = 11) populations were referenced most often.

### Overall Trends Over Time

**Figure 2.**
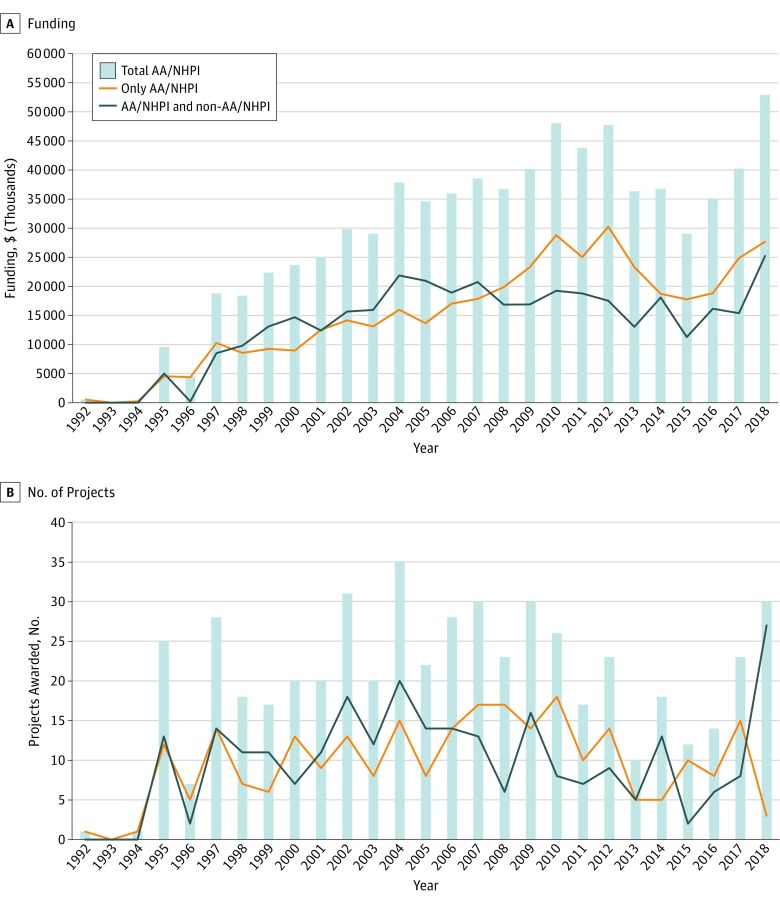
Funding for Research Grants and Number of Projects Focused on Asian American, Native Hawaiian, and Pacific Islander (AA/NHPI) Participants A, Dollar amounts for AA/NHPI clinical research grants over time. B, Number of new projects focusing on AA/NHPI populations awarded over time.

Figure 2A shows the dollar amounts for AA/NHPI clinical research grants over time. Total AA/NHPI dollars ($775 536 121) composed 0.17% of the overall NIH expenditures ($451 284 075 000) between 1992 and 2018, and 0.18% ($677 479 468) of the NIH research budget after 2000. In all, AA/NHPI-only and AA/NHPI plus non-AA/NHPI dollar amounts made up 0.10% ($362 547 841) and 0.09% ($314 931 627) of the total NIH expenditures after 2000, respectively (eTable 2 in the Supplement). There was a statistically significant positive trend in total AA/NHPI funding dollars between 1992 and 2018 (estimated dollar amount, $12 860 000; 95% CI, $9 215 000-$16 504 000; *P* < .001), but the proportion of the total NIH budget has only increased from 0.12% before 2000 to 0.18% after 2000, with no difference in funding before vs after 2000 (eTable 3 in the Supplement). Compared with funding before 2000, there was a statistically significant increase in funding amounts after 2000 for AA/NHPI-only projects (estimated dollar amount, $92 962 000; 95% CI, $13 756 000-$172 167 000; *P* = .02), while funding amounts for AA/NHPI plus non-AA/NHPI significantly decreased after 2000 (estimated dollar amount, −$115 265 000; 95% CI, −$183 009 000 to −$47 521 000; *P* < .001) (eTable 3 in the Supplement).

Total AA/NHPI research constituted 0.38% of the clinical research budget in 2018 and made up 0.36% of the NIH clinical research budget between 2008 and 2018 (eTable 4 in the Supplement). Spending for AA/NHPI-only projects made up 0.20% of the clinical budget in 2018 and had an average of 0.21% over the same period.

Figure 2B shows the number of new projects awarded over time. No statistically significant trend was observed in total new AA/NHPI projects awarded over time. There was a statistically significant increase in total AA/NHPI projects awarded after 2000 compared with projects awarded before 2000 (estimated number of projects, 9.89; 95% CI, 2.77-17.01 projects; *P* = .01) (eTable 5 in the Supplement). By grouping category, there was a statistically significant increase in projects awarded after 2000 for AA/NHPI only (estimated number of projects, 4.72; 95% CI, 0.65-8.79 projects; *P* = .02) and AA/NHPI plus non-AA/NHPI (estimated number of projects, 5.17; 95% CI, 0.06-10.27 projects; *P* = .05) groups.

## Discussion

Despite federal legislation and initiatives prioritizing data disaggregation and advancing health disparities research, we found that 0.17% of the overall NIH budget was allocated to 529 AA/NHPI-related clinical research projects over 2 decades. Our findings match the study by Ghosh^11^ that found AA/NHPI individuals represented 0.2% of the total health-related federal expenditures. Since the Ghosh study,^11^ the number and amount of AA/NHPI-only grants increased, but they still accounted for only one-fifth of 1% of NIH’s clinical research budget. The funding allocated to total AA/NHPI research remained less than one half of a percent for both the overall NIH budget and clinical research budget. Furthermore, there was a lack of diversity in the investment in terms of ICs and geographical regions. More than half of the research funding was concentrated in 3 ICs, consistent with the leading causes of death for AA/NHPI individuals in the United States.^35^ Funding was concentrated in California and Hawaii, corresponding to the top states of residence for AA/NHPI individuals.^36^ Similar to prior reports,^17,37^ negligible investment was seen toward states with the fastest-growing AA/NHPI populations. The current investment patterns, or lack thereof, could result in worsening health disparities in AA/NHPI populations because of prolonged disparate funding of health research.

Minimal increases in inclusion of racial/ethnic minority participants across all NIH research have been reported.^38^ Enrollment for AA participants in extramural NIH-defined phase 3 clinical trials increased from 2.5% (n = 7451) in 2011 to 12.1% (n = 19 172) in 2016, while NHPI participants decreased from 0.3% (n = 1011) to 0.2% (n = 271) during the same period.^38^ This increase in clinical trial participation by AA participants did not translate to increased data disaggregation. We found that AA/NHPI populations continue to be classified as a homogeneous group and there was unequal representation of AA subgroups. Dishearteningly, the decline in NHPI participants enrolled in NIH clinical trials matched our findings. Native Hawaiian and Pacific Islander participants were almost absent from the grants found in our search.

Treating AA/NHPI populations as homogeneous assumes cultural beliefs and experiences are the same, which could potentially influence and prolong misleading clinician stereotypes and unconscious biases about patients.^39^ Immigration- and acculturation-related factors influence English language proficiency, health and digital literacy, and preferences of clinical care and treatment.^39^ The magnitude of these factors varies across and within AA/NHPI subgroups, and differentially affects the generalizability of clinical research as well as the significance of downstream clinical applications. Disaggregated analyses have the potential to inform clinical and public health programs if significant differences are found between groups. Understanding cultural difference within AA/NHPI populations could emend the cycle of poor patient-clinician communication and patient health outcomes.^39^

Findings from our study have implications for future administrative and programmatic efforts, namely (1) intentional use of FOAs, (2) workforce diversity, (3) data disaggregation with data harmonization, and (4) access to data.

One reason for poor investment by NIH in AA/NHPI research could be that investigators are not submitting grant applications focusing on these populations. Our research could not asses the number of applications submitted that included AA/NHPI populations. However, we reviewed FOAs and we could differentiate funded grants that were in response to a specific FOA. Approximately half of grants in this analysis (393 of 529) were linked to a FOA. However, less than a quarter of FOAs specifically referenced AA/NHPI populations and only one-third of the FOAs referenced health disparities populations. Our finding suggests that FOAs need to be more intentional in referencing health disparate populations.

The low rate of funded research among AA/NHPI populations could be because of the low prevalence of AA/NHPI researchers. Our findings could not evaluate the race/ethnicity of principal investigators. However, only 5% of the total AA/NHPI funding was awarded to research career awards, training grants, and fellowships, but more than three-fifths of that amount was allocated to research project grants. Systematic challenges for minority investigators include mentoring differences, securing funding, and achieving academic tenure.^37,38,39,40,41^ For example, Ginther et al^42^ reported that Asian investigators were less likely to receive an R01 award on the first or second submission compared with their white counterparts. Enhancing investments in the research pipeline at the early career stages could improve the biomedical workforce diversity and could improve the number and success of funded research studies. In addition, improved workforce diversity could increase participation of AA/NHPI individuals in health research.^29,30,43^ Minority populations are more likely to understand and participate in research if they are able to identify or feel a connection with researchers.^44,45,46,47,48^

In our study, more AA/NHPI-only projects specified AA/NHPI subgroups compared with AA/NHPI plus non-AA/NHPI projects. This finding suggests that AA/NHPI-only projects may be prioritizing culturally appropriate inclusion and recruitment strategies as well as disaggregated data, while AA/NHPI plus non-AA/NHPI projects may focus more on studying a disease in general. More focused AA/NHPI research could improve the status of disaggregated data and accuracy of AA/NHPI health profiles, particularly if there is better harmonization of questionnaires and data collection protocols. For example, the Mediators of Atherosclerosis in South Asians Living in America (MASALA) study^49^ was modeled after the Multi-Ethnic Study of Atherosclerosis (MESA).^50^ Because MASALA and MESA had matching data collection protocols and collected disaggregated racial/ethnic data, the prevalence of diabetes is now comparable between specific subgroups of Asian individuals, Latino individuals, and white individuals.^50^

Federal agencies must provide public-use data files and reports by disaggregated race/ethnicity to improve transparency and evaluation of research priorities. Eliminating health disparities requires that there is sufficient AA/NHPI data to understand what is happening and to intervene in a meaningful way. Future studies should evaluate research rigor of funded projects, such as the proposed enrollment of racial/ethnic minority participants as comparison with enrollment reported in publications. Funding trends should be assessed to document whether they have kept pace with population growth and whether they are addressing and anticipating health disparities. Other federal agencies and philanthropic organizations should document their funding for AA/NHPI groups.

### Limitations

This study has limitations. First, this study summarized extramural funding awarded through the NIH and does not include other federal agencies or philanthropy. Search queries focused on research project grants, training, and fellowships as funding mechanisms because they focus on investigator-initiated research. The applicability of our review is limited to the project information available on NIH RePORTER, so relevant projects may have been excluded during the screening and eligibility protocol.

We do not know what proportion of NIH funding should be spent on racial/ethnic minority populations or what the research priorities should be. We assume that research funding in racial/ethnic minorities will lead to improved health outcomes because more disaggregated health data will improve our understanding of whether health disparities exist and result in evidence-based interventions for at-risk and high-risk AA/NHPI subgroups. Total AA/NHPI projects covered 10 of 11 *Healthy People 2020* health disparities areas that report AA/NHPI data. However, it is ambiguous how much investment and how many projects are needed to reach the *Healthy People 2020* goals, or when an arbitrary level of improved health has been achieved.

In addition, information on the inclusion of racial/ethnic subgroups in clinical research are not publicly available. Minority health and health disparities funding data are available on the RCDC summary table, but do not follow the standardized RCDC process for annual estimates. The clinical research budget line is only available starting in 2008. The proportions may be underestimated for the total NIH expenditures (ie, this denominator includes nonhuman subjects research) and overestimated for clinical research expenditures (ie, this denominator may not have included secondary data analyses). We also reported comparisons between total funding amounts and project means for AA/NHPI only and AA/NHPI plus non-AA/NHPI; however, the more meaningful comparisons may be comparisons with NIH total funding amounts by grant mechanism and mean funding per NIH project, respectively.

## Conclusions

We found disproportionate long-term investments from the NIH to eliminating health disparities in AA/NHPI populations. Inclusion for these underrepresented populations in the federal agenda and disaggregated data allows for more useful data to reveal the health status of AA/NHPI subgroups and helps to dispel the model minority stereotype. Overt direction from federal entities, dedicated federal funds for health disparities research, and parallel efforts to increase diversity in the biomedical workforce will be critical to advance health equity. This portfolio review can be used to address underlying systematic barriers and inform future health disparity research opportunities.

## Notes

Supplement. **eMethods. **NIH RePORTER Search Strategy **eTable 1.** Project Titles, Administering NIH Institute and Center, Funding Opportunity Announcement, Start Year, and Study Section Information for NIH-Funded Asian American, Native Hawaiian, & Pacific Islander Clinical Research, Federal Fiscal Years 1992-2018 (N=262 Unique FOAs) **eTable 2.** Proportions of Total NIH Expenditures by Asian American, Native Hawaiian, & Pacific Islander Grouping Category, Federal Fiscal Year 1992-2018 **eTable 3.** Estimates of Dollar Amounts Over Time for NIH-Funded Asian American, Native Hawaiian, & Pacific Islander Clinical Research, Federal Fiscal Year 1992-2018 **eTable 4.** Estimates of New Projects Awarded Over Time for NIH-Funded Asian American, Native Hawaiian, & Pacific Islander Clinical Research, Federal Fiscal Year 1992-2018 **eTable 5.** Proportions of Total NIH Clinical Research Expenditures by Asian American, Native Hawaiian, & Pacific Islander Grouping Category, Federal Fiscal Year 2008-2018
