# Supplementary material for: Trends in Clinical Research Including Asian American, Native Hawaiian, and Pacific Islander Participants Funded by the US National Institutes of Health, 1992 to 2018
Source: JAMA Netw Open. 2019 Jul 24;2(7):e197432. doi: 10.1001/jamanetworkopen.2019.7432 (PMC6659145; doi:10.1001/jamanetworkopen.2019.7432)
Supplement: Supplement. — eMethods. NIH RePORTER Search Strategy eTable 1. Project Titles, Administering NIH Institute and Center, Funding Opportunity Announcement, Start Year, and Study Section Information for NIH-Funded Asian American, Native Hawaiian, & Pacific Islander Clinical Research, Federal Fiscal Years 1992-2018 (N=262 Unique FOAs) eTable 2. Proportions of Total NIH Expenditures by Asian American, Native Hawaiian, & Pacific Islander Grouping Category, Federal Fiscal Year 1992-2018 eTable 3. Estimates of Dollar Amounts Over Time for NIH-Funded Asian American, Native Hawaiian, & Pacific Islander Clinical Research, Federal Fiscal Year 1992-2018 eTable 4. Estimates of New Projects Awarded Over Time for NIH-Funded Asian American, Native Hawaiian, & Pacific Islander Clinical Research, Federal Fiscal Year 1992-2018 eTable 5. Proportions of Total NIH Clinical Research Expenditures by Asian American, Native Hawaiian, & Pacific Islander Grouping Category, Federal Fiscal Year 2008-2018 [file jamanetwopen-2-e197432-s001.pdf]

Supplementary Online Content

Đoàn LN, Takata Y, Sakuma K-LK, Irvin VL. Trends in clinical research including Asian American, Native Hawaiian, and Pacific Islander participants funded by the US National Institutes of Health, 1992 to 2018. *JAMA Netw Open*. 2019;2(7):e197432. doi:10.1001/jamanetworkopen.2019.7432

eMethods. NIH RePORTER Search Strategy
eTable 1. Project Titles, Administering NIH Institute and Center, Funding Opportunity Announcement, Start Year, and Study Section Information for NIH-Funded Asian American, Native Hawaiian, & Pacific Islander Clinical Research, Federal Fiscal Years 1992-2018 (N=262 Unique FOAs)
eTable 2. Proportions of Total NIH Expenditures by Asian American, Native Hawaiian, & Pacific Islander Grouping Category, Federal Fiscal Year 1992-2018
eTable 3. Estimates of Dollar Amounts Over Time for NIH-Funded Asian American, Native Hawaiian, & Pacific Islander Clinical Research, Federal Fiscal Year 1992-2018
eTable 4. Estimates of New Projects Awarded Over Time for NIH-Funded Asian American, Native Hawaiian, & Pacific Islander Clinical Research, Federal Fiscal Year 1992-2018
eTable 5. Proportions of Total NIH Clinical Research Expenditures by Asian American, Native Hawaiian, & Pacific Islander Grouping Category, Federal Fiscal Year 2008-2018

This supplementary material has been provided by the authors to give readers additional information about their work.

© 2019 Đoàn LN et al. *JAMA Network Open.*

eMethods. NIH RePORTER Search Strategy

Text Search: ("Japanese" OR "Cambodian" OR "Vietnamese " OR "Thai" OR "Laotian" OR "Indonesian" OR "Malaysian" OR "Bangladeshi" OR "Pakistani" OR "Burmese" OR "Chinese" OR "Filipino" OR "Filipina" OR "Hawaiian" OR "Guamanian" OR "Samoan" OR "Chamorro" OR "Hmong" OR "Nepalese" OR "Bhutanese" OR "Sri Lankan" OR "Asian Indian" OR "South Asian" OR "Korean" OR "Asian American" OR "Pacific Islander" OR "Asian") NOT ("animal" OR "mouse%" OR "mosquito" OR "rhesus" OR "Aedes" OR "elegan" OR "bacteria" OR "leishmaniasis" OR "drosophila" OR "Alaskan" OR "genetic" OR "cellular" OR "genotype" OR "phenotype" OR "lymphocyte" OR "T cell" OR "vivax" OR "American Native" OR "Alaskan Native" OR "vitro" OR "falciparum" OR "Indian nation" OR "markers" OR "mice" OR "animal model") (Advanced); Search in: Projects Limit to: Project Abstracts; Project Title; Project Terms; AdminIC: NICHD; NCI; NCCIH; NCCAM; NCRR; NEI; NHLBI; NHGRI; NIAID; NIAMS; NIDCR; NIDDK; NIEHS; NIGMS; NIMH; NINDS; NINR; NIA; NIAAA; NIDCD; NIDA; NIMHD; NCMHD; OD; WH; ; Funding Mechanism: Non-SBIR/STTR; Research Centers; Other Research-Related; Training; Individual; Training; Institutional; Activity Code: P01 Program Projects; R01 Equivalents; DP2; R01; R23; R29; R37; RF1; R03; R21; M01; P20; P30; P50; U54; K01; K02; K07; K08; K23; K24; F31 Predoctoral; F32 Postdoctoral; FI2; Training Grants (Ts); State: All US States and Territories; Country(s): UNITED STATES AND TERRITORIES; Fiscal Year: 2018; 2017; 2016; 2015; 2014; 2013; 2012; 2011; 2010; 2009; 2008; 2007; 2006; 2005; 2004; 2003; 2002; 2001; 2000; 1999; 1998; 1997; 1996; 1995; 1994; 1993; 1992; 1991; 1990; 1989; 1988; 1987; 1986; 1985

© 2019 Đoàn LN et al. *JAMA Network Open.*

eTable1. Project titles, administering NIH institute and center, funding opportunity announcement, start year, and study section information for NIH-funded Asian American, Native Hawaiian, & Pacific Islander clinical research, federal fiscal years 1992-2018 (N=262 unique FOAs) [a]

Project Title	IC [b]	FOA [c]	FOA	Activity	Study Section [d]	Referenced AA/NHPI?
Racial and Ethnic Disparities Health Policy Model	NIA	PA-00-003	Mentored Clinical Scientist Development Award (K08)	K08	Behavior and Social Science of Aging Review Committee (NIA-S)	No
Asian Teens, Substance Dependence and Conduct Disorder	NIDA	PA-00-003	Mentored Clinical Scientist Development Award (K08)	K08	Training and Career Development Subcommittee (NIDA-K)	No
Metabolic Syndrome and Atherosclerosis in South Asians	NHLBI	PA-00-004	Mentored Patient-Oriented Research Career Development Award (K23)	K23	Special Emphasis Panel (ZHL1-CSR-M (O1))	No
Culture-Specific, Multimedia Cardiovascular Disease Education For Asian Indians	NHLBI	PA-00-004	Mentored Patient-Oriented Research Career Development Award (K23)	K23	Special Emphasis Panel (ZHL1-CSR-M (F1))	No
Bridging Menopause: Experiences/Clinical Encounters	NICHD	PA-00-004	Mentored Patient-Oriented Research Career Development Award (K23)	K23	Population Sciences Subcommittee (CHHD-W)	No
Cognitive-Behavior Therapy For Khmer Refugees	NIMH	PA-00-004	Mentored Patient-Oriented Research Career Development Award (K23)	K23	Special Emphasis Panel (ZMH1-NRB-G (10))	No
Culturally Sensitive Treatment For Depressed Asians	NIMH	PA-00-004	Mentored Patient-Oriented Research Career Development Award (K23)	K23	Special Emphasis Panel (ZMH1-NRB-G (13))	No
Measuring Neighborhood: Multiethnic Older Adults	NIA	PA-00-019	Mentored Research Scientist Development Award (K01)	K01	Behavior and Social Science of Aging Review Committee (NIA-S)	No - Health Disparity Populations
Ecological Factors and Drug Use of Native Hawaiian Youth	NIDA	PA-00-019	Mentored Research Scientist Development Award (K01)	K01	Training and Career Development Subcommittee (NIDA-K)	No - Health Disparity Populations

© 2019 Đoàn LN et al. *JAMA Network Open.*

Racial Identity and Psychosocial Consequences	NIMH	PA-00-019	Mentored Research Scientist Development Award (K01)	K01	Special Emphasis Panel (ZRG1-RPHB-4 (01)S)	No - Health Disparity Populations
Asian Immigrant Cultural Adjustment and Mental Health	NIMH	PA-00-019	Mentored Research Scientist Development Award (K01)	K01	Special Emphasis Panel (ZMH1-CRB-J (06))	No - Health Disparity Populations
Designing Culturally and Socially Valid Interventions	NIMH	PA-00-019	Mentored Research Scientist Development Award (K01)	K01	Interventions Research Review Committee (ITV)	No - Health Disparity Populations
Risk Factors For Problem Behaviors of Asian Youth	NIMH	PA-00-019	Mentored Research Scientist Development Award (K01)	K01	Risk, Prevention and Health Behavior 1 (RPHB-1)	No - Health Disparity Populations
Ee and Stigma Among Chinese-Americans With Schizophrenia	NIMH	PA-00-019	Mentored Research Scientist Development Award (K01)	K01	Behavioral Genetics and Epidemiology Study Section (BGES)	No - Health Disparity Populations
Ethnogeriatric Cultural Competence: Chronic Confusion	NINR	PA-00-019	Mentored Research Scientist Development Award (K01)	K01	Special Emphasis Panel (NRRC (25))	No - Health Disparity Populations
Health & Well Being of Older Asian Indian Immigrants	NIA	PA-00-053	NIA Pilot Research Grant Program	R03	Special Emphasis Panel (ZAG1-ZIJ-1 (O1))	No
Elderly Immigrants: the Experience of Asian Indians	NIA	PA-00-053	NIA Pilot Research Grant Program	R03	Special Emphasis Panel (ZAG1-ZIJ-1 (O1))	No
Predictors of Adaptation Among Indian-Americans	NICHD	PA-00-069	NIH Predoctoral Fellowship Awards for Minority Students (F31)	F31	Special Emphasis Panel (ZRG1-SSS-C (29)L)	Yes- AAs/ NHPIs

© 2019 Đoàn LN et al. *JAMA Network Open.*

Cognitive Behavioral Intervention in Diabetes Self-Mgt.	NINR	PA-00-113	Diabetes Self-Management in Minority Populations	R01	Nursing Research Study Section (NURS)	Yes-AAs/NHPIs
Type 2 Diabetes Primary Prevention For At Risk Girls	NIDDK	PA-01-017	Physical Activity and Obesity Across Chronic Diseases	R01	Special Emphasis Panel (ZRG1-SNEM-1 (01)S)	No
Diabetes and Cardiovascular Disease in Filipina Women	NIDDK	PA-01-031	NIDDK Small Grants for Underrepresented Investigators	R03	Diabetes, Endocrinology and Metabolic Diseases B Subcommittee (DDK-B)	No - Health Disparity Populations
Ethnic Minority Children in Public Mental Health	NIMH	PA-01-044	Research on Emergency Medical Services for Children	R01	Services Research Review Committee (SRV)	No - Health Disparity Populations
Sexuality, HIV/Drug in 3 Groups of Asian/Gay/Bi Men/Msm	NIDA	PA-01-096	Behavioral, Social, Mental Health, and Substance Abuse Research with Diverse Populations	R01	Special Emphasis Panel (ZRG1-AARR-7 (01))	No - Health Disparity Populations
Club Drugs, Dance Events, and Asian-American Youth	NIDA	PA-02-043	Social and Cultural Dimensions of Health	R01	Special Emphasis Panel (ZRG1-HOP-A (03)M)	No - Health Disparity Populations
Cognitive Consensus in Cross-Cultural Competence	NIMH	PA-02-043	Social and Cultural Dimensions of Health	R01	Services Research Review Committee (SRV)	No - Health Disparity Populations
A Multiethnic Internet Study on Menopausal Symptoms	NINR	PA-02-043	Social and Cultural Dimensions of Health	R01	Special Emphasis Panel (ZRG1-NSCF (01)S)	No - Health Disparity Populations
South Asians Engaging in Hypertension Awareness & Treatment (Saehat)	NINR	PA-02-134	Community-Partnered Interventions to Reduce Health Disparities	R21	Community-Level Health Promotion Study Section (CLHP)	Yes-AAs/NHPIs

Ethnic Disparities in Diabetes Complications	NIDDK	PA-02-165	Race/Ethnic Disparities in the Incidence of Diabetes Complications	R01	Health Services Organization and Delivery Study Section (HSOD)	Yes-AAs/NHPIs
Testing Segmented Assimilation Theory With Add Health	NICHD	PA-03-057	Social and Demographic Studies of Race and Ethnicity in the United States	R01	Social Sciences and Population Studies Study Section (SSPS)	Yes-AAs/NHPIs
Us Minority Migration and Metropolitan Change	NICHD	PA-03-057	Social and Demographic Studies of Race and Ethnicity in the United States	R01	Social Sciences and Population Studies Study Section (SSPS)	Yes-AAs/NHPIs
Education and Health Among Racial Groups	NICHD	PA-03-057	Social and Demographic Studies of Race and Ethnicity in the United States	R01	Special Emphasis Panel (ZRG1-HOP-J (90)S)	Yes-AAs/NHPIs
Depression and Motivation: Self-Objectification	NIMH	PA-03-067	Ruth L. Kirschstein National Research Service Awards for Individual Postdoctoral Fellows (F32)	F32	Special Emphasis Panel (ZRG1-F11 (20)L)	No
Ethnicity and Pregnancy Outcomes in New York City	NICHD	PA-03-107	NIH Exploratory/Developmental Research Grant Award (R21)	R21	Special Emphasis Panel (ZRG1-HOP-B (90)S)	No
Go Early: Promoting Mammography Use Among Korean American Women	NINR	PA-03-107	NIH Exploratory/Developmental Research Grant Award (R21)	R21	Nursing Science: Adults and Older Adults Study Section (NSAA)	No
Aging, Acculturation, and Health: Korean American Elders	NIA	PA-03-108	NIH Small Research Grant Program (R03)	R03	Social Psychology, Personality and Interpersonal Processes Study Section (SPIP)	No
Gangs, Gender and Drug Sales: A Qualitative Study	NIDA	PA-03-139	Women, Gender Differences and Drug Abuse	R01	Community Influences on Health Behavior Study Section (CIHB)	No
Cultural Variation in Affect Valuation	NIMH	PA-03-169	Basic and Translational Research in Emotion	R01	Social Psychology, Personality and Interpersonal Processes Study Section (SPIP)	No
Columbia-Ghi Consortium For Claims-Based Cancer Studies	NCI	PA-04-012	Cancer Surveillance Using Health Claims-Based Data System	R21	Epidemiology of Cancer	No

© 2019 Đoàn LN et al. *JAMA Network Open.*

					Study Section (EPIC)	
Population Genetics & Drug Dependence in Asian Isolates	NIDA	PA-04-032	Ruth L. Kirschstein National Research Service Awards for Individual Predoctoral Fellows (F31)	F31	Training and Career Development Subcommittee (NIDA-K)	No
Culture and Ethnic Variations in Breast Cancer Treatment	NCI	PA-04-034	Exploratory Grants for Behavioral Research in Cancer Control	R21	Special Emphasis Panel (ZRG1-CIHB (01)Q)	No - Health Disparity Populations
Understand Disparities in Cervical Cancer Survival	NCI	PA-04-034	Exploratory Grants for Behavioral Research in Cancer Control	R21	Epidemiology of Cancer Study Section (EPIC)	No - Health Disparity Populations
Fathers Experience With Pediatric End-of-Life Care	NINR	PA-04-057	Improving Care for Dying Children and their Families	R01	Nursing Science: Children and Families Study Section (NSCF)	No
Studies in Children With Digestive Disorders	NIDDK	PA-04-107	Midcareer Investigator Award in Patient-Oriented Research (K24)	K24	Special Emphasis Panel (ZDK1-GRB-S (O3)M)	No
Organizational Change Toward HIV Involvement in Immigrant Religious Organizations	NICHD	PA-04-115	Religious Organizations and HIV	R01	Special Emphasis Panel (ZRG1-RPHB-B (50)R)	No
Aspects of Bone Quality Among Chinese American Women	NIAMS	PA-05-143	Mentored Patient-Oriented Research Career Development Award (K23)	K23	Arthritis and Musculoskeletal and Skin Diseases Special Grants Review Committee (AMS)	No
Tobacco Dependence Treatments For Asian Americans	NIDA	PA-05-143	Mentored Patient-Oriented Research Career Development Award (K23)	K23	Training and Career Development Subcommittee (NIDA-K)	No
Methadone Population Pharmacokinetics: Treatment Outcome in Hmong and Non-Hmong	NIDA	PA-05-143	Mentored Patient-Oriented Research Career Development Award (K23)	K23	Training and Career Development Subcommittee (NIDA-K)	No
A Cross-Cultural, Patient-Centered Approach For	NIMH	PA-05-143	Mentored Patient-Oriented Research	K23	Mental Health Services in	No

Assessing Quality of Adhd Care			Career Development Award (K23)		Non-Specialty Settings (SRNS)	
HIV/Stis Risk Behaviors Among Chinese, Korean, and Vietnamese Women	NIMH	PA-06-001	Mentored Research Scientist Development Award (K01)	K01	Behavioral and Social Science Approaches to Preventing HIV/AIDS Study Section (BSPH)	No
Nutritional Risk Factors For Type 2 Diabetes in the Multiethnic Cohort	NIDDK	PA-06-151	Secondary Analyses in Obesity, Diabetes, Digestive and Kidney Diseases (R21)	R21	Kidney, Nutrition, Obesity and Diabetes Study Section (KNOD)	No
Visceral Adiposity and Mortality in Japanese Americans	NIDDK	PA-06-151	Secondary Analyses in Obesity, Diabetes, Digestive and Kidney Diseases (R21)	R21	Cardiovascular and Sleep Epidemiology Study Section (CASE)	No
Teenagers, Families, and Well-Being	NICHD	PA-06-180	NIH Small Research Grant Program (Parent R03)	R03	Population Sciences Subcommittee (CHHD-W)	No
Health Impacts of Hurricane Katrina on Vietnamese-American New Orleanians	NICHD	PA-06-181	NIH Exploratory/Developmental Research Grant Program (Parent R21)	R21	Special Emphasis Panel (ZRG1-HOP-B (90)S)	No
Mental Health Literacy Among Korean American Elders	NIMH	PA-06-181	NIH Exploratory/Developmental Research Grant Program (Parent R21)	R21	Social Psychology, Personality and Interpersonal Processes Study Section (SPIP)	No
CBPR on Active Living and Recovery For Racial/Ethnic Groups With Mental Illness	NIMH	PA-06-181	NIH Exploratory/Developmental Research Grant Program (Parent R21)	R21	Community Influences on Health Behavior Study Section (CIHB)	No
A Culturally-Relevant Risk Reduction Intervention With Native Hawaiian Mothers	NINR	PA-06-181	NIH Exploratory/Developmental Research Grant Program (Parent R21)	R21	Nursing Science: Children and Families Study Section (NSCF)	No
Screening For Colortectal Cancer in an Asian Community Center	NCI	PA-06-351	Exploratory Grants for Behavioral Research in Cancer Control (R21)	R21	Special Emphasis Panel (ZRG1-HOP-T (90))	No - Health Disparity Populations

© 2019 Đoàn LN et al. *JAMA Network Open.*

Beliefs About the Pap Smear and Cervical Cancer Screening Among Vietnamese Women	NCI	PA-06-351	Exploratory Grants for Behavioral Research in Cancer Control (R21)	R21	Community Influences on Health Behavior Study Section (CIHB)	No - Health Disparity Populations
an Individually-Tailored Mammography Screening Intervention For Chinese Americans	NCI	PA-06-351	Exploratory Grants for Behavioral Research in Cancer Control (R21)	R21	Psychosocial Risk and Disease Prevention Study Section (PRDP)	No - Health Disparity Populations
Breast & Cervical Cancer Screening Among Hmong: Social, Cultural & System Factor	NCI	PA-06-351	Exploratory Grants for Behavioral Research in Cancer Control (R21)	R21	Community Influences on Health Behavior Study Section (CIHB)	No - Health Disparity Populations
Cultural Impact on Adaptation Between Chinese and White Breast Cancer Survivors	NCI	PA-06-351	Exploratory Grants for Behavioral Research in Cancer Control (R21)	R21	Behavioral Medicine, Interventions and Outcomes Study Section (BMIO)	No - Health Disparity Populations
the Influence of the Built Environment on Outcomes After Breast Cancer	NCI	PA-06-405	Studies of Energy Balance and Cancer in Humans (R21)	R21	Epidemiology of Cancer Study Section (EPIC)	No
Translational Research: Applying Drug Prevention to Obesity Prevention	NICHD	PA-06-415	School-Based Interventions to Prevent Obesity (R01)	R01	Community-Level Health Promotion Study Section (CLHP)	No
Stress and Coping in Minority Adolescents: an Internet-Based Daily Diary Study	NICHD	PA-06-481	Ruth L. Kirschstein National Research Service Awards for Individual Predoctoral Fellowships (F31) to Promote Diversity in Health-Related Research	F31	Special Emphasis Panel (ZRG1-HOP-T (29)L)	No
Alcohol and Cultural Factors and the Underreporting of Rape	NIAAA	PA-07-002	Ruth L. Kirschstein National Research Service Awards for Individual Predoctoral Fellows (F31)	F31	Epidemiology, Prevention and Behavior Research Review Subcommittee (AA-2)	No
Anxiety and Help Seeking Behaviors	NIMH	PA-07-002	Ruth L. Kirschstein National Research Service Awards for	F31	Special Emphasis Panel	No

© 2019 Đoàn LN et al. *JAMA Network Open.*

			Individual Predoctoral Fellows (F31)		(ZRG1-F16-T (20)L)	
Better Breast and Cervical Cancer Control For Korean American Women	NCI	PA-07-045	Social and Cultural Dimensions of Health (R01)	R01	Community-Level Health Promotion Study Section (CLHP)	No
Luteal Adjuvant Oophorectomy in Asian Breast Cancer	NCI	PA-07-070	Research Project Grant (Parent R01)	R01	Epidemiology of Cancer Study Section (EPIC)	No
HR-QOL in Colorectal Cancer Survivors With Stomas	NCI	PA-07-070	Research Project Grant (Parent R01)	R01	Nursing Science: Adults and Older Adults Study Section (NSAA)	No
Effects of A Smart Growth Community on Prevention of Family Obesity Risk	NCI	PA-07-070	Research Project Grant (Parent R01)	R01	Community-Level Health Promotion Study Section (CLHP)	No
A RCT to Promote Mammography Adherence Among Chinese Immigrant Women	NCI	PA-07-070	Research Project Grant (Parent R01)	R01	Nursing Science: Adults and Older Adults Study Section (NSAA)	No
Project F-Eat: Families and Eating and Activity in Teens	NHL BI	PA-07-070	Research Project Grant (Parent R01)	R01	Community Influences on Health Behavior Study Section (CIHB)	No
Association of Sleep Disorders With Cardiovascular Health Across Ethnic Groups	NHL BI	PA-07-070	Research Project Grant (Parent R01)	R01	Special Emphasis Panel (ZRG1-PSE-A (02)M)	No
Mediators of Atherosclerosis in South Asians Living in America	NHL BI	PA-07-070	Research Project Grant (Parent R01)	R01	Cardiovascular and Sleep Epidemiology Study Section (CASE)	No
Ethnic Identity & Psychological Adjustment Among African, Asian & Latino Youth	NIC HD	PA-07-070	Research Project Grant (Parent R01)	R01	Social Psychology, Personality and Interpersonal Processes Study Section (SPIP)	No
Heterosexual Men's Perspectives on Sexual Behavior and Sexual Risk Taking	NIC HD	PA-07-070	Research Project Grant (Parent R01)	R01	Behavioral and Social Science Approaches to Preventing HIV/AIDS	No

© 2019 Đoàn LN et al. *JAMA Network Open.*

					Study Section (BSPH)	
Psychosocial Benefits of Ethnic Diversity in Urban Middle Schools	NICHD	PA-07-070	Research Project Grant (Parent R01)	R01	Special Emphasis Panel (ZRG1-RPHB-E (02)M)	No
Cultural Effects on Stress, Coping, and Symptom Expression	NIMH	PA-07-070	Research Project Grant (Parent R01)	R01	Community Influences on Health Behavior Study Section (CIHB)	No
Telepsychiatry & Culturally Sensitive Treatment of Depressed Asian Americans	NIMH	PA-07-070	Research Project Grant (Parent R01)	R01	Mental Health Services in Non-Specialty Settings (SRNS)	No
Risk and Protective Factors For Depression Among Racial Groups	NIMH	PA-07-070	Research Project Grant (Parent R01)	R01	Social Psychology, Personality and Interpersonal Processes Study Section (SPIP)	No
Power and Risk Among Gay Couples	NIMH	PA-07-070	Research Project Grant (Parent R01)	R01	Behavioral and Social Science Approaches to Preventing HIV/AIDS Study Section (BSPH)	No
Immigration Effects on Substance Abuse, Mental Health and Treatment Gaps	NIDA	PA-07-082	Risk Factors for Psychopathology Using Existing Data Sets (R01)	R01	Behavioral Genetics and Epidemiology Study Section (BGES)	No - Health Disparity Populations
Predictors of Change Profiles in Studies of Adolescent Substance Abusers	NIDA	PA-07-082	Risk Factors for Psychopathology Using Existing Data Sets (R01)	R01	Health Services Research Subcommittee (NIDA-F)	No - Health Disparity Populations
CBPR Approach in Cancer-Screening Promotion	NCI	PA-07-106	Ruth L. Kirschstein National Research Service Awards for Individual Predoctoral Fellowships (F31) to Promote Diversity in Health-Related Research	F31	Special Emphasis Panel (ZRG1-HOP-Z (29)L)	No
Ideal Affect and Health Decision Making Across the Lifespan	NIA	PA-07-106	Ruth L. Kirschstein National Research Service Awards for	F31	Special Emphasis Panel	No

			Individual Predoctoral Fellowships (F31) to Promote Diversity in Health-Related Research		(ZRG1-RPHB-K (29)L)	
Poverty, Discrimination, and Achievement Motivation in Urban Minority Adolescents	NIC HD	PA-07-107	Ruth L. Kirschstein National Research Service Awards (NRSA) for Individual Postdoctoral Fellows (F32)	F32	Special Emphasis Panel (ZRG1-F11-B (20)L)	No
The Mental Health and Well-Being of Adolescent Children of Cambodian Refugees	NIM H	PA-07-312	Mental Health Consequences of Violence and Trauma (R01)	R01	Psychosocial Development, Risk and Prevention Study Section (PDRP)	No
Identifying Disparities in Type 2 Diabetes Among Asian Americans: the Pan Asian C	NID DK	PA-07-388	Identifying and Reducing Diabetes and Obesity Related Health Disparities within Healthcare Systems (R01)	R01	Kidney, Nutrition, Obesity and Diabetes Study Section (KNOD)	No - Healt h Dispa rity Popul ations
Club Drugs, Dance Events, and Asian-American Youth	NID A	PA-07-409	Health Research with Diverse Populations (R01)	R01	Special Emphasis Panel (ZRG1-HOP-A (03)M)	No - Healt h Dispa rity Popul ations
Lay Health Workers and Colorectal Cancer Screening Among Chinese Americans	NCI	PA-08-074	Community Participation in Research (R01)	R01	Community-Level Health Promotion Study Section (CLHP)	No - Healt h Dispa rity Popul ations
A Pap Test Intervention to Enhance Decision Making Among Pacific Islander Women	NCI	PA-08-074	Community Participation in Research (R01)	R01	Community-Level Health Promotion Study Section (CLHP)	No - Healt h Dispa rity Popul ations
Healthy Immigrant Families: Working Together to Move More and to Eat Well	NHL BI	PA-08-074	Community Participation in Research (R01)	R01	Community-Level Health Promotion Study Section (CLHP)	No - Healt h Dispa rity Popul ations
Alcohol Use Among Asian American Adolescents & Young	NIA AA	PA-08-169	Secondary Analysis of Existing Alcohol	R03	Special Emphasis Panel	No

© 2019 Đoàn LN et al. *JAMA Network Open.*

Adults: Do Subgroups Differ?			Epidemiology Data (R03)		(ZRG1-PSE-H (80)S)	
Mobile Phone Text Messaging Intervention For Cervical Cancer Screening	NCI	PA-09-130	Exploratory Grants for Behavioral Research in Cancer Control (R21)	R21	Special Emphasis Panel (ZRG1-HDEP-D (01)Q)	No - Health Disparity Populations
A Cross-National Perspective in Migrant Health	NICHD	PA-09-163	NIH Small Research Grant Program (Parent R03)	R03	Population Sciences Subcommittee (CHHD-W)	No
Stress and Coping Model of Alcohol Use Among Treatment-Seeking Asian American Adu	NIAAA	PA-09-208	Ruth L. Kirschstein National Research Service Awards for Individual Predoctoral Fellows (F31)	F31	Clinical, Treatment and Health Services Research Review Subcommittee (AA-3)	No
Aging, Emotion Regulation, Social Relationships, and Health Processes	NIA	PA-09-209	Ruth L. Kirschstein National Research Service Awards for Individual Predoctoral Fellowships (F31) to Promote Diversity in Health-Related Research	F31	Special Emphasis Panel (ZRG1-BBBP-J (29)L)	No - Health Disparity Populations
Hawaii Asian and Pacific Islander Diabetes Study	NIDDK	PA-09-262	Health Disparities in NIDDK Diseases (R01)	R01	Health Disparities and Equity Promotion Study Section (HDEP)	Yes- AAs/ NHPIs
Stress and Moral Judgment Variation Across Contexts Among Immigrant Asian Youth	NICHD	PA-10-064	NIH Small Research Grant Program (Parent R03)	R03	Population Sciences Subcommittee (CHHD-W)	No
Stress and Health Among Sexual Minority Immigrants	NICHD	PA-10-064	NIH Small Research Grant Program (Parent R03)	R03	Health, Behavior, and Context Subcommittee (CHHD-M)	No
Increasing HPV Vaccine Uptake in A Low Income Ethnic Minority Population	NCI	PA-10-067	Research Project Grant (Parent R01)	R01	Community-Level Health Promotion Study Section (CLHP)	No
Culture, Social Support & Quality of Life: Asian American Breast Cancer Survivors	NCI	PA-10-067	Research Project Grant (Parent R01)	R01	Health Disparities and Equity Promotion Study Section (HDEP)	No

© 2019 Đoàn LN et al. *JAMA Network Open.*

Lay Health Worker Model to Reduce Liver Cancer Disparities in Asian Americans	NCI	PA-10-067	Research Project Grant (Parent R01)	R01	Health Disparities and Equity Promotion Study Section (HDEP)	No
Multiethnic Study of Breast Arterial Calcium Gradation and CVD	NHL BI	PA-10-067	Research Project Grant (Parent R01)	R01	Cardiovascular and Sleep Epidemiology Study Section (CASE)	No
Bedside Interpreter Intervention, Hospital Outcomes of Older LEP Patients	NIA	PA-10-067	Research Project Grant (Parent R01)	R01	Special Emphasis Panel (ZRG1-NRCS-B (01)S)	No
Translating a Heart Disease Lifestyle Intervention Into the Community	NHL BI	PA-10-069	NIH Exploratory Developmental Research Grant Program (Parent R21)	R21	Community-Level Health Promotion Study Section (CLHP)	No
Chinese Health and Aging Project: Building Support Through Community Engagement	NIA	PA-10-069	NIH Exploratory Developmental Research Grant Program (Parent R21)	R21	Community Influences on Health Behavior Study Section (CIHB)	No
A Family Intervention to Reduce Smoking Among Chinese and Vietnamese Men	NID A	PA-10-069	NIH Exploratory Developmental Research Grant Program (Parent R21)	R21	Special Emphasis Panel (ZRG1-RPIA-K (09)F)	No
Developmental Pathways to Substance Use Across European & Asian American Youth	NIA AA	PA-10-108	Ruth L. Kirschstein National Research Service Awards for Individual Predoctoral Fellows (Parent F31)	F31	Epidemiology, Prevention and Behavior Research Review Subcommittee (AA-2)	No
Adolescent AOD Use Trajectories: the Role of Race and Ethnicity	NIA AA	PA-11-016	Epidemiology and Prevention in Alcohol Research (R01)	R01	Community Influences on Health Behavior Study Section (CIHB)	No
Coping With Discrimination: Alcohol Use in Filipino/A American Young Adults	NIA AA	PA-11-111	Ruth L. Kirschstein National Research Service Awards for Individual Predoctoral Fellows (Parent F31)	F31	Epidemiology, Prevention and Behavior Research Review Subcommittee (AA-2)	No
Predoctoral Fellowship in Public and Community Health- Successful Aging Among ASI	NIA	PA-11-112	Ruth L. Kirschstein National Research Service Awards for Individual Predoctoral Fellowships to Promote Diversity in Health-Related	F31	Special Emphasis Panel (ZRG1-F16-B (20)L)	Yes-AAs/ NHPI s

			Research (Parent F31 - Diversity)			
Disparities in Health and Academics of Sexual Minorities in Urban Middle Schools	NIC HD	PA-11-113	Ruth L. Kirschstein National Research Service Awards (NRSA) for Individual Postdoctoral Fellows (Parent F32)	F32	Special Emphasis Panel (ZRG1-F16-L (20)L)	No
Do Barriers to Transplantation Impact Liver Cancer Survival in Asians?	NCI	PA-11-164	the Effect of Racial and Ethnic Discrimination/Bias on Health Care Delivery (R03)	R03	Health Disparities and Equity Promotion Study Section (HDEP)	Yes-AAs/ NHPI s
Engaging Immigrants in Preventive Parenting Interventions	NIC HD	PA-11-194	Mentored Patient-Oriented Research Career Development Award (Parent K23)	K23	Health, Behavior, and Context Subcommittee (CHHD-M)	No
Midcareer Award in Patient-Oriented Community-Academic Partnered Aging Research	NIA	PA-11-195	Midcareer Investigator Award in Patient-Oriented Research (Parent K24)	K24	Clinical Aging Review Committee (NIA-C)	No
State Medical Marijuana Laws and Marijuana Use and Consequences Since 2004	NID A	PA-11-230	Epidemiology of Drug Abuse (R01)	R01	Social Sciences and Population Studies B Study Section (SSPB)	No
Culture, Family Process, and Developmental Outcomes in Asian American Youth	NIC HD	PA-11-260	Research Project Grant (Parent R01)	R01	Psychosocial Development, Risk and Prevention Study Section (PDRP)	No
Reducing Workplace Chemical Exposures in Nail Salon Workers Using an Owner-to-Wor	NIE HS	PA-11-260	Research Project Grant (Parent R01)	R01	Community-Level Health Promotion Study Section (CLHP)	No
Examining the Geographies of Immigrant Sex Work: an Exploratory Study of HIV Risk	NIC HD	PA-11-261	NIH Exploratory/Developmental Research Grant Program (Parent R21)	R21	Behavioral and Social Science Approaches to Preventing HIV/AIDS Study Section (BSPH)	No
Diabetes and Obesity: Associations With Cognition, Neuropathology, and Longevity	NIA	PA-11-262	NIH Small Research Grant Program (Parent R03)	R03	Neurological, Aging and Musculoskeletal Epidemiology (NAME)	No
the Development and Evaluation of the Ho'ouna Pono Drug Prevention Curriculum	NID A	PA-11-311	Drug Abuse Prevention Intervention Research (R01)	R01	Health Disparities and Equity Promotion	Yes-AAs/ NHPI s

					Study Section (HDEP)	
Identity Stress and Health in Three Cohorts of LGB Individuals	NICHD	PA-12-111	Research on the Health of LGBTI Populations [R01]	R01	Health Disparities and Equity Promotion Study Section (HDEP)	No - Health Disparity Populations
Identity Stress and Health in Three Cohorts of LGB Individuals	NICHD	PA-12-111	Research on the Health of LGBTI Populations [R01]	R01	Health Disparities and Equity Promotion Study Section (HDEP)	No - Health Disparity Populations
Identity Stress and Health in Three Cohorts of LGB Individuals	NICHD	PA-12-149	Research Supplements to Promote Diversity in Health-Related Research (Admin Supp)	R01	Health Disparities and Equity Promotion Study Section (HDEP)	Yes-AAs/NHPIs
Promoting Healthy Seafood Choices in Asian Communities	NIEHS	PA-12-153	Research to Action: Assessing and Addressing Community Exposures to Environmental Contaminants (R01)	R01	Community Influences on Health Behavior Study Section (CIHB)	No - Health Disparity Populations
A Biobehavioral Model of Diabetes Risk in Chinese Immigrants	NIDDK	PA-13-183	Addressing Health Disparities in NIDDK Diseases (R01)	R01	Social Psychology, Personality and Interpersonal Processes Study Section (SPIP)	Yes-AAs/NHPIs
A Web-Based Intervention to Promote Follow-Up Care Communication and Functional Status of Chinese Immigrant Breast Cancer Survivors	NCI	PA-13-288	Behavioral and Social Science Research on Understanding and Reducing Health Disparities (R21)	R21	Special Emphasis Panel (ZRG1-NRCS-V (08))	Yes-AAs/NHPIs
Engaging Pacific Islander Perspectives on Mental Illness and Mental Health Services	NIMH	PA-13-288	Behavioral and Social Science Research on Understanding and Reducing Health Disparities (R21)	R21	Mental Health Services Research Committee (SERV)	Yes-AAs/NHPIs
Joy Luck Academy: A Culturally Sensitive Social Support Intervention	NCI	PA-13-292	Behavioral and Social Science Research on Understanding and Reducing Health Disparities (R01)	R01	Health Disparities and Equity Promotion Study Section (HDEP)	Yes-AAs/NHPIs

© 2019 Đoàn LN et al. *JAMA Network Open.*

to Enhance Breast Cancer Survivorship of Asian Americans	NCI	PA-13-292	Behavioral and Social Science Research on Understanding and Reducing Health Disparities (R01)	R01	Special Emphasis Panel (ZRG1-HDM-J (02))	Yes-AAs/NHPIs
Limited English Proficiency, Health, and Healthcare Among Older Immigrants	NIA	PA-13-292	Behavioral and Social Science Research on Understanding and Reducing Health Disparities (R01)	R01	Health Disparities and Equity Promotion Study Section (HDEP)	Yes-AAs/NHPIs
Building Community Capacity For Disability Prevention For Minority Elders	NIA	PA-13-292	Behavioral and Social Science Research on Understanding and Reducing Health Disparities (R01)	R01	Health Disparities and Equity Promotion Study Section (HDEP)	Yes-AAs/NHPIs
Adaptive Intervention to Maximize Colorectal Screening in Safety Net Populations	NCI	PA-13-302	Research Project Grant (Parent R01)	R01	Community-Level Health Promotion Study Section (CLHP)	No
Molecular Mechanism of Hypertrophic Cardiomyopathy in Populations of South Asian Descendants	NHLBI	PA-13-302	Research Project Grant (Parent R01)	R01	Myocardial Ischemia and Metabolism Study Section (MIM)	No
Mediators of Atherosclerosis in South Asians Living in America	NHLBI	PA-13-302	Research Project Grant (Parent R01)	R01	Cardiovascular and Sleep Epidemiology Study Section (CASE)	No
Cardiovascular Disease Among Asians and Pacific Islanders (Casper)	NHLBI	PA-13-302	Research Project Grant (Parent R01)	R01	Special Emphasis Panel (ZRG1-PSE-K (90)S)	No
Femur Fracture Outcomes Associated With Bisphosphonate Use	NIA	PA-13-302	Research Project Grant (Parent R01)	R01	Skeletal Biology Development and Disease Study Section (SBDD)	No
Lifecourse Health, Cerebral Pathology and Ethnic Disparities in Dementia	NIA	PA-13-302	Research Project Grant (Parent R01)	RF1	Special Emphasis Panel (ZRG1-PSE-P (02))	No
A Family-Focused Intervention For Asian American Male Smokers	NIDA	PA-13-302	Research Project Grant (Parent R01)	R01	Special Emphasis Panel (ZRG1-RPIA-N (09)F)	No
Lifestyle and Medication Management to Lower Diabetes Risk in Severe Mental Illness	NIDDK	PA-13-302	Research Project Grant (Parent R01)	R01	Community-Level Health Promotion Study Section (CLHP)	No

© 2019 Đoàn LN et al. *JAMA Network Open.*

Medicare Policy Effects on Mental Health Care Disparities	NIMHD	PA-13-302	Research Project Grant (Parent R01)	R01	Health Disparities and Equity Promotion Study Section (HDEP)	No
Obesity and Caries in Young South Asian Children: A Common Risk Factor Approach	NIMHD	PA-13-302	Research Project Grant (Parent R01)	R01	Community-Level Health Promotion Study Section (CLHP)	No
Culture and Caregiving Need For Chinese Elderly With Cognitive Impairment	NINR	PA-13-302	Research Project Grant (Parent R01)	R01	Nursing and Related Clinical Sciences Study Section (NRCS)	No
the Effects of Discrimination and Sleep Disturbance on Health Among Asian Youth	NIMHD	PA-13-303	NIH Exploratory/Developmental Research Grant Program (Parent R21)	R21	Psychosocial Development, Risk and Prevention Study Section (PDRP)	No
Development of A Group Prenatal Care Intervention to Address Maternal and Child Ncd Risk in American Samoa	NINR	PA-13-303	NIH Exploratory/Developmental Research Grant Program (Parent R21)	R21	Nursing and Related Clinical Sciences Study Section (NRCS)	No
Integrated Community-Clinical Linkage Model to Promote Weight Loss Among South Asians With Pre-Diabetes	NIDDK	PA-13-352	Translational Research to Improve Diabetes and Obesity Outcomes (R01)	R01	Health Disparities and Equity Promotion Study Section (HDEP)	No
A Culturally-Relevant Approach to Reducing Dementia Caregiver Stress in an Underserved Population	NIA	PA-14-044	Mentored Research Scientist Development Award (Parent K01)	K01	Behavior and Social Science of Aging Review Committee (NIA-S)	No
Development of A Healthcare Navigation Intervention	NICHD	PA-14-049	Mentored Patient-Oriented Research Career Development Award (Parent K23)	K23	Health, Behavior, and Context Subcommittee (CHHD-M)	No
Building Community Capacity For Disability Prevention For Minority Elders	NIA	PA-14-078	Change of Grantee Organization (Type 7 Parent)	R01	Health Disparities and Equity Promotion Study Section (HDEP)	No
Psychometric Evaluation of Ahimsa Scale	NIAAA	PA-14-190	Epidemiology and Prevention in Alcohol Research (R01)	R01	Addiction Risks and Mechanisms	No - Health

© 2019 Đoàn LN et al. *JAMA Network Open.*

					Study Section (ARM)	Disparity Populations
Disadvantage and Sociocultural Modifiers in Asian American Drinking	NIAAA	PA-14-337	Secondary Analyses of Existing Alcohol Epidemiology Data (R03)	R03	Health Disparities and Equity Promotion Study Section (HDEP)	No
Improving Diabetes Risk Assessment and Screening in Minority-Predominant Community Health Center Patients	NIDDK	PA-15-169	Secondary Analyses in Obesity, Diabetes and Digestive and Kidney Diseases (R21)	R21	Health Disparities and Equity Promotion Study Section (HDEP)	No
Cardiovascular Disease Among Asians and Pacific Islanders (Casper)	NHLBI	PA-15-322	Research Supplements to Promote Diversity in Health-Related Research (Admin Supp)	R01	Special Emphasis Panel (ZRG1-PSE-K (90)S)	Yes-AAs/NHPIs
the Kaholo Project: Preventing Cardiovascular Disease in Native Hawaiians	NHLBI	PA-15-322	Research Supplements to Promote Diversity in Health-Related Research (Admin Supp)	R01	Special Emphasis Panel (ZRG1-HDM-V (51))	Yes-AAs/NHPIs
Risk of Venous Thromboembolism Following Diagnosis and Treatment of Multiple Myeloma: Differences By Race	NHLBI	PA-16-036	Improving Outcomes in Cancer Treatment-Related Cardiotoxicity (R21)	R21	Cancer, Heart, and Sleep Epidemiology B Study Section (CHSB)	No - Health Disparity Populations
Community Translation of the South Asian Healthy Lifestyle Intervention (Saheli)	NHLBI	PA-16-160	NIH Research Project Grant (Parent R01)	R01	Special Emphasis Panel (CLTR (JA))	No
Next Generation Association Studies of Adiposity in Samoans Enhanced By A Samoan-Specific Whole Genome Sequence Reference Panel	NHLBI	PA-16-160	NIH Research Project Grant (Parent R01)	R01	Kidney, Nutrition, Obesity and Diabetes Study Section (KNOD)	No
Impact of Multilevel Risk and Resilience Factors on Cardiovascular Health in Racially/Ethnically Diverse Men and Women	NHLBI	PA-16-160	NIH Research Project Grant (Parent R01)	R01	Health Disparities and Equity Promotion Study Section (HDEP)	No
Population-Level Interventions and Community Environment Effects on Child Obesity Disparities	NHLBI	PA-16-160	NIH Research Project Grant (Parent R01)	R01	Community-Level Health Promotion Study Section (CLHP)	No

© 2019 Đoàn LN et al. *JAMA Network Open.*

Bilingual and Socio-Emotional Development in Dual Language Learners	NIC HD	PA-16-160	NIH Research Project Grant (Parent R01)	R01	Psychosocial Development, Risk and Prevention Study Section (PDRP)	No
Impact of Heritage Language on Bilingual Children's Path to English Literacy	NIC HD	PA-16-160	NIH Research Project Grant (Parent R01)	R01	Language and Communicatio n Study Section (LCOM)	No
Culturally Adapted Multilevel Decision Support Navigation Trial to Reduce Colorectal Cancer Disparity Among At-Risk Asian American Primary Care Patients	NIM HD	PA-16-160	NIH Research Project Grant (Parent R01)	R01	Health Disparities and Equity Promotion Study Section (HDEP)	No
Acculturation, Family Relations, and Mental Health of Chinese Older Adults in the United States	NIA	PA-16-161	NIH Exploratory/Develop mental Research Grant Program (Parent R21)	R21	Social Sciences and Population Studies A Study Section (SSPA)	No
Developing A Prevention Model of Alcohol Use Disorder For Pacific Islander Young Adults	NIA AA	PA-16-161	NIH Exploratory/Develop mental Research Grant Program (Parent R21)	R21	Epidemiology, Prevention and Behavior Research Review Subcommittee (AA-2)	No
Healthy Bodies, Healthy Souls	NIM HD	PA-16-161	NIH Exploratory/Develop mental Research Grant Program (Parent R21)	R21	Community-Level Health Promotion Study Section (CLHP)	No
Work Hour Patterns and Diabetes Disparities	NIM HD	PA-16-161	NIH Exploratory/Develop mental Research Grant Program (Parent R21)	R21	Special Emphasis Panel (ZRG1-HDEP-C (91))	No
Understanding Self- and Family-Management in HIV-Positive Asian Americans in New York	NIM HD	PA-16-162	NIH Small Research Grant Program (Parent R03)	R03	Special Emphasis Panel (ZRG1-AARR-N (02)M)	No
Improving Breast Cancer Risk Prediction With Composite Measures of Obesity and Body Fat Distribution	NCI	PA-16-175	Exploratory Grants in Cancer Epidemiology and Genomics Research (R21)	R21	Cancer, Heart, and Sleep Epidemiology A Study Section (CHSA)	No - Healt h Dispa rity Popul ations
Medication Adherence, Health Literacy and Cultural Health Beliefs in A Mass. Chc	NHL BI	PA-16-285	Change of Grantee Organization (Type 7 Parent)	R01	Special Emphasis Panel (ZRG1-HDM-X (56)R)	No
Psychometric Evaluation of Ahimsa Scale	NIA AA	PA-16-288	Research Supplements to Promote Diversity	R01	Addiction Risks and	Yes-AAs/

© 2019 Đoàn LN et al. *JAMA Network Open.*

			in Health-Related Research (Admin Supp)		Mechanisms Study Section (ARM)	NHPIs
Race/Ethnic Specific Association Between Anthropometry, Liver Fat and Incident Type 2 Diabetes: Evidence From the Multi-Ethnic Study of Atherosclerosis	NIDDK	PA-16-308	Ruth L. Kirschstein National Research Service Award Individual Predoctoral Fellowship to Promote Diversity in Health-Related Research (Parent F31 - Diversity)	F31	Special Emphasis Panel (ZDK1-GRB-2 (O1)L)	Yes-AAs/NHPIs
Temporal Milestones During Childhood: Identification of A Maturational Spurt	NICHD	PA-16-309	Ruth L. Kirschstein National Research Service Award (Nrsa) Individual Predoctoral Fellowship (Parent F31)	F31	Special Emphasis Panel (ZRG1-F10B-B (20)L)	No
Cardiometabolic Health in Adolescents of South Asian Ancestry - the Charisma Study	NIDDK	PA-17-021	Addressing Health Disparities in NIDDK Diseases (R01)	R01	Clinical and Integrative Diabetes and Obesity Study Section (CIDO)	Yes-AAs/NHPIs
Linking State Policies to Latino and Asian American Immigrant Health Care Access	NIMHD	PA-17-041	Addressing the Etiology of Health Disparities and Health Advantages Among Immigrant Populations (R01)	R01	Health Disparities and Equity Promotion Study Section (HDEP)	Yes-AAs/NHPIs
Immigrant Enclaves: Conferring Health Advantages Or Creating Health Disparities in Chinese Immigrants?	NIMHD	PA-17-041	Addressing the Etiology of Health Disparities and Health Advantages Among Immigrant Populations (R01)	R01	Community Influences on Health Behavior Study Section (CIHB)	Yes-AAs/NHPIs
Phthalate Exposure and Gender-Related Development	NIEHS	PA-18-676	Research on the Health of Women of Understudied, Underrepresented and Underreported (U3) Populations – an ORWH FY18 Administrative Supplement (Admin Supp Clinical Trial Optional)	R01		Yes-AAs/NHPIs
Asian-Indian Adolescents--Generational Conflict/Drug Use	NIDA	PA-91--08		R03	Epidemiology and Prevention Research Subcommittee (NIDA-G)	
Methodology in Prevention of Hepatocellular Carcinoma	NCI	PA-91--87		K07	Clinical Cancer Education Committee (CEC)	

© 2019 Đoàn LN et al. *JAMA Network Open.*

Psychopathology and Environments in Drug Abuse	NIDA	PA-91-101		K02	Special Emphasis Panel (SRCD-B)	
Collaborative Study of Ovarian Cancer in Two Risk Groups	NCI	PA-92--27		R01	Epidemiology and Disease Control Subcommittee 2 (EDC-2)	
National Research Center on Asian American Mental Health	NIMH	PA-92-104	Minority Mental Health Research Centers	R01	Special Emphasis Panel (SRCM-B)	Yes-AAs/NHPIs
Age and Illness Management in Later Life	NIA	PA-93-076	Health and Effective Functioning in the Middle and Later Years	R01	Human Development and Aging Subcommittee 2 (HUD-2)	Yes-AAs/NHPIs
Age, Ethnicity, and the Chronically Ill Unisured	NIA	PA-93-076	Health and Effective Functioning in the Middle and Later Years	R01	Special Emphasis Panel (ZRG1-SNEM-1 (01)S)	Yes-AAs/NHPIs
Alcohol, Gangs & Violence--A Preprevention Expoloration	NIAAA	PA-93-095		R01	Epidemiology and Prevention Subcommittee (ALCP-2)	
Alcohol, Violence and Female Gangs--A Qualitative Study	NIAAA	PA-93-095		R01	Community Prevention and Control Study Section (CPC)	
Perceptions of Friendships Among Urban Adolescents	NIMH	PA-94--02	Behavioral Science Track Award for Rapid Transition	R03	Special Emphasis Panel (SRCM-M)	No
Chinese American Families of Schizophrenia	NIMH	PA-94-002	Behavioral Science Track Award for Rapid Transition	R03	Special Emphasis Panel (SRCM-M)	No
Cultural Influences on Relived Emotions	NIMH	PA-94-002	Behavioral Science Track Award for Rapid Transition	R03	Special Emphasis Panel (SRCM-M)	No
Culture and Family Reactions to Schizophrenia	NIMH	PA-94-002	Behavioral Science Track Award for Rapid Transition	R03	Special Emphasis Panel (SRCM-M)	No
Sleep During the Perimenopause in A Multi-Ethnic Cohort	NIA	PA-94-086		R01	Special Emphasis Panel (ZAG1-ZIJ-4 (O3))	
Minority Predoctoral Fellowship Program	NICHD	PA-95-029	Predoctoral Fellowship Awards for Minority Students	F31	Special Emphasis Panel (ZRG2-PSF (02)L)	Yes-AAs/NHPIs
Immigrant Second Generation in Metropolitan New York	NICHD	PA-95-036	Research on U.S. Immigration	R01	Special Emphasis Panel (ZRG2-BEM (02)M)	No - Health Disparity Populations

© 2019 Đoàn LN et al. *JAMA Network Open.*

Models of the Occurrence and Timing of Naturalization	NICHD	PA-95-036	Research on U.S. Immigration	R01	Social Sciences and Population Study Section (SSP)	No - Health Disparity Populations
Race/Ethnicity in School Based/Mental Health Services	NIMH	PA-95-049	Mentored Research Scientist Development Award	K01	Services Research Review Committee (SER)	No
Psychopathology and Environments in Drug Abuse	NIDA	PA-95-050	Independent Scientist Award	K02	Special Emphasis Panel (SRCD-B)	No
Contingencies of Self-Esteem	NIMH	PA-95-050	Independent Scientist Award	K02	Special Emphasis Panel (ZRG1-RPHB-1 (02)M)	No
HIV-Related Behaviors Among Transgenders of Color	NIDA	PA-95-057	HIV Risk Behaviors, Determinants and Consequences	R01	Special Emphasis Panel (ZRG1 (01))	No - Health Disparity Populations
HIV Risk Reduction Among Asian Women	NIDA	PA-95-083	Women's HIV Risk and Protective Behaviors	R01	Special Emphasis Panel (ZRG1-AARR-8 (01)S)	No - Health Disparity Populations
Asian American Mental Health	NIMH	PA-96-007	Research on the Mental Health of Minority Populations	R01	Special Emphasis Panel (ZMH1-CRB-C (01))	Yes-AAs/NHPIs
Mortality Among Asian American Elderly	NIA	PA-96-023	Pilot Project Research Grant Program for the NIA	R03	Behavior and Social Science of Aging Review Committee (NIA-S)	No
Migration Effects on Health of Working Age Vietnamese	NICHD	PA-96-025	Nichd Small Grants Program	R03	Special Emphasis Panel (CHHD (DB)-G)	No
Activating Multiethnic Youth For Smoking Prevention	NCI	PA-97-055	Priorities in Behavioral Research in Cancer Prevention and Control	R01	Community Prevention and Control Study Section (CPC)	No
Cultural Differences in Self-Reports of Well-Being	NIMH	PA-98-031	Methodology and Measurement in the Behavioral and Social Sciences	R01	Special Emphasis Panel (ZRG1-RPHB-4 (01)S)	Yes-AAs/NHPIs

© 2019 Đoàn LN et al. *JAMA Network Open.*

Treating Chinese Smokers With Interactive Expert Systems	NIDA	PA-98-052	Mentored Patient-Oriented Research Career Development Award	K23	Training and Career Development Subcommittee (NIDA-K)	No
Midcareer Mentoring and Dementia Caregiving Research	NINR	PA-98-053	Midcareer Investigator Award in Patient-Oriented Research	K24	Special Emphasis Panel (NRRC (15))	No
Older Patient-Physician-Alternative Healer Relationships	NIA	PA-98-059	Health-Care Encounters Between Elderly Patients, Physicians, and Other Care Providers	R01	Special Emphasis Panel (ZRG1-SNEM-1 (01)S)	No - Health Disparity Populations
Racial and Neighborhood Disparities in Infant Health	NICHD	PA-98-098		R01	Special Emphasis Panel (ZRG1-SSS-D (03)M)	
A Writing Program For Multiethnic Breast Cancer Patients	NCI	PA-99-163	Exploratory Grants for Behavioral Research in Cancer Control	R21	Special Emphasis Panel (ZRG1-RPHB-3 (01)S)	No - Health Disparity Populations
Women's Use of Alternative Medicine: A Multiethnic Study	NCCAM	PAR-00-023	Predoctoral Research Training in Complementary and Alternative Medicine	F31	Special Emphasis Panel (ZAT1-CP (02))	No
Pregnancy Hormonal Profile/Biomarker Breast Cancer Risk	NCI	PAR-00-025	Cancer Prevention Research Small Grant Program	R03	Special Emphasis Panel (ZCA1-SRRB-Q (J1))	No
Ethnicity, Gender, and Measuring Adolescent Depression	NIMH	PAR-00-119	Behavioral Science Track Award for Rapid Transition (B/Start)	R03	Special Emphasis Panel (ZMH1-BST-W (01))	No
Social Networks Among Drug-Using Ethnic Minority Youth	NIDA	PAR-01-014		R03	Special Emphasis Panel (ZDA1-JXP-R (09))	
Faith-Based Breast Cancer Screening Intervention	NCI	PAR-01-135	Cancer Prevention, Control, Behavioral and Population Sciences Career Development Award (K07)	K07	Subcommittee G - Education (NCI-G)	No
Marc U*Star At California State University, Northridge	NIGMS	PAR-02-033	Marc Undergraduate Student Training in Academic Research (U-Star) Program	T34	Minority Programs Review Subcommittee A (MPRC-A)	Yes-AAs/NHPIs
Marc U*Star Scholars Program At the	NIGMS	PAR-02-033	Marc Undergraduate Student Training in	T34	Minority Programs	Yes-AAs/

© 2019 Đoàn LN et al. *JAMA Network Open.*

University of Houston Downtown			Academic Research (U-Star) Program		Review Subcommittee A (MPRC-A)	NHPIs
Marc Biomedical Research and Training Program At the University of Arizona	NIGMS	PAR-02-033	Marc Undergraduate Student Training in Academic Research (U-Star) Program	T34	Minority Programs Review Subcommittee A (MPRC-A)	Yes-AAs/NHPIs
Quality of Life in Multiethnic Testis Cancer Survivors	NCI	PAR-02-037	Small Grants Program for Behavioral Research in Cancer Control	R03	Special Emphasis Panel (ZCA1-SRRB-D (J2))	No - Health Disparity Populations
Breast Density, Igf-I, & Prolactin, in Four Populations	NCI	PAR-03-010	Small Grants Program for Cancer Epidemiology	R03	Special Emphasis Panel (ZCA1-SRRB-Q (O1))	No
Social and Built Environment and Race/Ethnic Variations in Cancer Outcomes	NCI	PAR-03-010	Small Grants Program for Cancer Epidemiology	R03	Special Emphasis Panel (ZCA1-SRRB-Q (J1))	No
Culture, Age and Affect Valuation	NIA	PAR-03-056	NIA Pilot Research Grant Program	R03	Behavior and Social Science of Aging Review Committee (NIA-S)	No - Health Disparity Populations
Use and Correlates of Cam Among Racial/Ethnic Minorities	NCCAM	PAR-03-102	Secondary Analysis of Data on Cam Use in Minority Populations	R03	Special Emphasis Panel (ZAT1-DB (12))	Yes-AAs/NHPIs
Use of Herbs and Dietary Supplements in Four Ethnicities	NCCAM	PAR-03-102	Secondary Analysis of Data on Cam Use in Minority Populations	R21	Special Emphasis Panel (ZAT1-DB (12))	Yes-AAs/NHPIs
Cam Use in Asians & Pacific Islanders	NCCAM	PAR-03-102	Secondary Analysis of Data on Cam Use in Minority Populations	R21	Special Emphasis Panel (ZAT1-JH (06))	Yes-AAs/NHPIs
Herbal Medicine Use in Minority Populations in the Us	NCCAM	PAR-03-102	Secondary Analysis of Data on Cam Use in Minority Populations	R21	Special Emphasis Panel (ZAT1-DB (12))	Yes-AAs/NHPIs
Cam Use and Access to Care By Asian Pacific Islanders	NCCAM	PAR-03-102	Secondary Analysis of Data on Cam Use in Minority Populations	R21	Special Emphasis Panel (ZAT1-DB (12))	Yes-AAs/NHPIs
Minorities and Cam Use: A Health Services Perspective	NCCAM	PAR-03-102	Secondary Analysis of Data on Cam Use in Minority Populations	R21	Special Emphasis Panel (ZAT1-JH (06))	Yes-AAs/NHPIs

© 2019 Đoàn LN et al. *JAMA Network Open.*

Access and Utilization of Cam By Minorities	NCCAM	PAR-03-102	Secondary Analysis of Data on Cam Use in Minority Populations	R21	Special Emphasis Panel (ZAT1-LD (06))	Yes-AAs/NHPIs
Youths' Emotion Regulation: Mediators and Moderators	NIMH	PAR-04-010	Behavioral Science Track Award for Rapid Transition (B/Start)	R03	Special Emphasis Panel (ZMH1-BST-I (02))	No
Better Breast Health For Korean American Women	NCI	PAR-04-020	Small Grants for Behavioral Research in Cancer Control	R03	Special Emphasis Panel (ZCA1-SRRB-Q (J2))	No - Health Disparity Populations
Developing an Expectancy Challenge For Diverse Teens	NCI	PAR-04-020	Small Grants for Behavioral Research in Cancer Control	R03	Special Emphasis Panel (ZCA1-SRRB-K (O1))	No - Health Disparity Populations
Promoting Adherence to Mammography Use in Chinese Ameri*	NCI	PAR-04-020	Small Grants for Behavioral Research in Cancer Control	R03	Special Emphasis Panel (ZCA1-SRRB-4 (M1))	No - Health Disparity Populations
Sport: Sports to Prevent Obesity Randomized Trial	NIDDK	PAR-04-082	Small Clinical Grants in Digestive Diseases, Nutrition and Obesity	R03	Special Emphasis Panel (ZDK1-GRB-8 (O1)S)	No
Health Literacy Intervention For Korean Americans With Hpb	NHLBI	PAR-04-116	Understanding and Promoting Health Literacy (R01)	R01	Special Emphasis Panel (ZRG1-RPHB-B (51)R)	No - Health Disparity Populations
Culture and Cancer Literacy Among Immigrant Women	NCI	PAR-04-117	Understanding and Promoting Health Literacy (R03)	R03	Special Emphasis Panel (ZRG1-RPHB-J (50)R)	No - Health Disparity Populations
Involving Communities in Multicultural Network: Access	NHLBI	PAR-05-026	Community Participation in Research	R21	Special Emphasis Panel (ZRG1-HOP-S (50)R)	No - Health Disparity Populations

Physical Activity & Nutrition Among Pacific Islander Youth: an Exploratory Study	NICHD	PAR-05-026	Community Participation in Research	R21	Special Emphasis Panel (ZRG1-HOP-U (90)S)	No - Health Disparity Populations
Maternal Influences & Adolescent Sexual Behaviors: Aapi & Caucasian	NINR	PAR-05-091	Ruth L. Kirschstein National Research Service Award (NRSA) for Individual Predoctoral Fellows (F31) in Nursing Research	F31	Special Emphasis Panel (NRRC (38))	No
Chronic Psychological Stress and Arterial Stiffness in Korean Americans	NINR	PAR-05-091	Ruth L. Kirschstein National Research Service Award (NRSA) for Individual Predoctoral Fellows (F31) in Nursing Research	F31	Special Emphasis Panel (NRRC (44))	No
Principal Research Core	NIMH	PAR-05-161	Advanced Centers for Innovation in Services and Intervention Research (ACISIR)	P30	Special Emphasis Panel (ZMH1-ERB-B (01))	No - Health Disparity Populations
Cancer Control Dissemination to Asian Americans	NCI	PAR-06-039	Dissemination and Implementation Research in Health (R01)	R01	Special Emphasis Panel (ZRG1-HOP-S (51)R)	No - Health Disparity Populations
Cancer Risks in Multi-Ethnic Carriers of Unclassified Brca1 Variants	NCI	PAR-06-294	Small Grants Program for Cancer Epidemiology (R03)	R03	Special Emphasis Panel (ZCA1-SRRB-D (O1))	No
Isoflavones, Equol Producing Status, and Breast Density in Us Chinese Women	NCI	PAR-06-294	Small Grants Program for Cancer Epidemiology (R03)	R03	Special Emphasis Panel (ZCA1-SRRB-D (M1))	No
A Pooled Analysis of Mammographic Density and Breast Cancer Risk	NCI	PAR-06-294	Small Grants Program for Cancer Epidemiology (R03)	R03	Special Emphasis Panel (ZCA1-SRRB-D (J1))	No
Groundwork For A Study of Non-Smoking-Associated Lung Cancer in ASIanpacific Isla	NCI	PAR-06-294	Small Grants Program for Cancer Epidemiology (R03)	R03	Special Emphasis Panel (ZCA1-SRRB-D (J1))	No
the Role of Churches in Cancer Prevention and Control Among Korean Americans	NCI	PAR-06-381	Cancer Prevention, Control, Behavioral, and Population Sciences Career	K07	Subcommittee J - Career Development (NCI-J)	No

© 2019 Đoàn LN et al. *JAMA Network Open.*

			Development Award (K07)			
Family Communication in Coping With Ethnic Minority Breast Cancer Survivors	NCI	PAR-06-458	Small Grants for Behavioral Research in Cancer Control (R03)	R03	Special Emphasis Panel (ZCA1-SRLB-H (J1))	No - Health Disparity Populations
Career Development Project	NIDCR	PAR-06-505	Specialized Programs of Research Excellence (Spores) in Human Cancer for the Year 2007 (P50)	P50	Special Emphasis Panel (ZCA1)	No
Dissemination Through Community Health Centers Serving Diverse Populations	NCI	PAR-06-521	Dissemination and Implementation Research in Health (R21)	R21	Special Emphasis Panel (ZRG1-HOP-X (50)R)	No - Health Disparity Populations
Reducing Disparities in Colorectal Cancer Screening in Vietnamese Americans	NCI	PAR-07-283	Community Participation in Research (R01)	R01	Special Emphasis Panel (ZRG1-HOP-S (52)R)	No - Health Disparity Populations
CBPR With Immigrant Chinese With Diabetes	NINR	PAR-07-283	Community Participation in Research (R01)	R01	Special Emphasis Panel (ZRG1-HOP-S (52)R)	No - Health Disparity Populations
Minority Access to Research Careers	NIGMS	PAR-07-337	MARC Undergraduate Student Training in Academic Research, Institutional National Research Service Award (NRSA) Research Training Grant (T34)	T34	Minority Programs Review Subcommittee A (MPRC-A)	Yes-AAs/NHPIs
Ucsc-Marc U*Star Program	NIGMS	PAR-07-337	MARC Undergraduate Student Training in Academic Research, Institutional National Research Service Award (NRSA) Research Training Grant (T34)	T34	Minority Programs Review Subcommittee B (MPRC-B)	Yes-AAs/NHPIs
Evaluation of A Hepatitis B Outreach Intervention For Cambodians	NCI	PAR-07-379	Behavioral and Social Science Research on Understanding and	R01	Special Emphasis Panel (ZRG1-RPHB-K (50)R)	Yes-AAs/NHPIs

			Reducing Health Disparities (R01)			
Impact of Contextual Factors on Disparities in Breast Cancer Risk and Survival	NCI	PAR-07-379	Behavioral and Social Science Research on Understanding and Reducing Health Disparities (R01)	R01	Special Emphasis Panel (ZRG1-RPHB-K (50)R)	Yes-AAs/NHPIs
A Patient-Centered Strategy to Reduce Diabetes Disparities in Chinese Americans	NINR	PAR-07-380	Behavioral and Social Science Research on Understanding and Reducing Health Disparities (R21)	R21	Special Emphasis Panel (ZRG1-RPHB-K (50)R)	Yes-AAs/NHPIs
Vaccine-Preventable Cancer Behavior Measurement Among Southeast Asian Americans	NCI	PAR-08-213	Methodology and Measurement in the Behavioral and Social Sciences (R21)	R21	Special Emphasis Panel (ZRG1-BBBP-D (52)R)	Yes-AAs/NHPIs
Measurement of Tobacco Use Among South Asians in the U.S.	NCI	PAR-08-213	Methodology and Measurement in the Behavioral and Social Sciences (R21)	R21	Special Emphasis Panel (ZRG1-HDM-Q (54)R)	Yes-AAs/NHPIs
Efficacy of Measurements For Api Populations in Us Based Samples and Surveys	NICHD	PAR-08-213	Methodology and Measurement in the Behavioral and Social Sciences (R21)	R21	Special Emphasis Panel (ZRG1-BBBP-D (52)R)	Yes-AAs/NHPIs
Smoking Cessation Across Ethnic Groups in the Us	NCI	PAR-09-003	Small Grants for Behavioral Research in Cancer Control (R03)	R03	Special Emphasis Panel (ZCA1-SRLB-Y (O3))	No - Health Disparity Populations
Hypertension and Health Literacy in Vietnamese Americans	NINR	PAR-09-227	Ruth L. Kirschstein National Research Service Award (NRSA) for Individual Predoctoral Fellows in Nursing Research (F31)	F31	Special Emphasis Panel (NRRC (46))	No - Health Disparity Populations
Stereotype Threat and Risk-Taking Propensity Among Asian Americans Young Adults	NIDA	PAR-09-239	Behavioral Science Track Award for Rapid Transition (B/START) (R03)	R03	Special Emphasis Panel (ZDA1-GXM-A (05)S)	No
Examining the Relations of Cigarette and Alcohol Use Across Emerging Adulthood	NIDA	PAR-09-239	Behavioral Science Track Award for Rapid Transition (B/START) (R03)	R03	Special Emphasis Panel (ZDA1-MXS-M (04)S)	No
Adapting Patient Navigation to Promote Cancer Screening in Chicagos Chinatown	NCI	PAR-10-038	Dissemination and Implementation Research in Health (R01)	R01	Dissemination and Implementation Research in Health Study Section (DIRH)	No - Health Disparity Populations

Marc Biomedical Research and Training Program At the University of Arizona	NIGMS	PAR-10-119	MARC Undergraduate Student Training in Academic Research (U-STAR) National Research Service Award (NRSA) Institutional Research Training Grant (T34	T34	Minority Programs Review Subcommittee A (MPRC-A)	Yes-AAs/NHPIs
Medication Adherence, Health Literacy and Cultural Health Beliefs in A Mass. Chc	NHLBI	PAR-10-133	Understanding and Promoting Health Literacy (R01)	R01	Special Emphasis Panel (ZRG1-HDM-X (56)R)	No - Health Disparity Populations
Health Literacy and Cancer Screening Disparities in Asian American Populations	NCI	PAR-10-134	Understanding and Promoting Health Literacy (R03)	R03	Special Emphasis Panel (ZRG1-RPHB-P (50)R)	No - Health Disparity Populations
Social and Cultural Influences on Cardiovascular Risk in South Asians	NHLBI	PAR-10-136	Behavioral and Social Science Research on Understanding and Reducing Health Disparities (R01)	R01	Health Disparities and Equity Promotion Study Section (HDEP)	Yes-AAs/NHPIs
Epidemiology of Psychological Distress in A Chinese Aging Population	NIA	PAR-10-136	Behavioral and Social Science Research on Understanding and Reducing Health Disparities (R01)	R01	Health Disparities and Equity Promotion Study Section (HDEP)	Yes-AAs/NHPIs
Tai Chi For Treating Major Depressive Disorder in Underserved Chinese Americans	NCCAM	PAR-10-137	Behavioral and Social Science Research on Understanding and Reducing Health Disparities	R21	Health Disparities and Equity Promotion Study Section (HDEP)	Yes-AAs/NHPIs
HPV Vaccination in the Cambodian Community	NCI	PAR-10-137	Behavioral and Social Science Research on Understanding and Reducing Health Disparities	R21	Health Disparities and Equity Promotion Study Section (HDEP)	Yes-AAs/NHPIs
Developing Neighborhood Archetypes For Understanding Disparities in Cancer	NCI	PAR-10-137	Behavioral and Social Science Research on Understanding and Reducing Health Disparities	R21	Health Disparities and Equity Promotion Study Section (HDEP)	Yes-AAs/NHPIs
Physical Activity Behavior Among Midlife	NINR	PAR-10-211	Ruth L. Kirschstein National Research Service Awards for	F31	Special Emphasis Panel (NRRC (49))	No

© 2019 Đoàn LN et al. *JAMA Network Open.*

South Asian Indian Immigrants			Individual Predoctoral Fellows in Nursing Research (F31)			
University of Hawaii Cancer Center CCSG	NCI	PAR-11-005	Cancer Center Support Grants (CCSGs) for NCI-Designated Cancer Centers (P30)	P30	Subcommittee A - Cancer Centers (NCI-A)	No - Health Disparity Populations
Relationships Between Discrimination and the Health of South Asian Americans	NINR	PAR-11-117	NINR Ruth L. Kirschstein National Research Service Awards for Individual Predoctoral Fellows in Nursing Research (F31)	F31	Special Emphasis Panel (NRRC (55))	No
the Kaholo Project: Preventing Cardiovascular Disease in Native Hawaiians	NHLBI	PAR-11-346	Interventions for Health Promotion and Disease Prevention in Native American Populations (R01)	R01	Special Emphasis Panel (ZRG1-HDM-V (51))	Yes-AAs/NHPIs
Investigating the Cause of Racial/Ethnic Disparity in Hepatocellular Cancer Risk	NCI	PAR-12-039	Small Grants Program for Cancer Epidemiology (R03)	R03	Special Emphasis Panel (ZCA1-SRLB-D (J1))	No - Health Disparity Populations
Risk Factors For Breast Cancer Subtypes in Racial/Ethnic Minorities	NCI	PAR-12-039	Small Grants Program for Cancer Epidemiology (R03)	R03	Special Emphasis Panel (ZCA1-SRB-H (M1))	No - Health Disparity Populations
Outreach Core	NCI	PAR-12-055	Limited Competition: Comprehensive Partnerships to Advance Cancer Health Equity (CPACHE) (U54)	U54	Special Emphasis Panel (ZCA1-SRLB-Y)	Yes-AAs/NHPIs
Obesity and Igf-Axis Activation in Native Hawaiian Women With Breast Cancer	NCI	PAR-12-094	Exploratory/Developmental Grants Program for Basic Cancer Research in Cancer Health Disparities (R21)	R21	Special Emphasis Panel (ZRG1-OBT-J (55)R)	No - Health Disparity Populations
Biologic Basis of Disparity in Liver Cancer Survival Among Asian Americans	NCI	PAR-12-094	Exploratory/Developmental Grants Program for Basic Cancer Research in Cancer Health Disparities (R21)	R21	Special Emphasis Panel (ZRG1-OBT-M (55)R)	No - Health Disparity

						Populations
Southeast Asian Women's Health Project	NCI	PAR-12-144	Nci Small Grants Program for Cancer Research (Nci Omnibus R03)	R03	Special Emphasis Panel (ZCA1-SRLB-B (O1))	No
Behavioral Intervention to Reduce Breast Cancer Disparity in Underserved Koreans	NCI	PAR-12-145	Nci Exploratory/Developmental Research Grant Program (Nci Omnibus R21)	R21	Special Emphasis Panel (ZCA1-SRLB-B (M1))	No
Project 3: Linking Maternal Obesity and offspring Cancer Risks Through Integrati	NIGMS	PAR-13-243	Renewal of Centers of Biomedical Research Excellence [COBRE](P20)	P20	Special Emphasis Panel (ZGM1-TWD-7 (C2))	No
Research Project (Rp) 3 - Katrina Impacts on Vietnamese Americans in New Orleans (Kativa Nola)	NICHD	PAR-13-257	Nichd Program Project Grant (P01)	P01	Special Emphasis Panel (ZHD1-DSR-M (MV))	No
Administrative Core	NIMHD	PAR-13-279	Limited Competition: NIMHD Research Centers in Minority Institutions Infrastructure for Clinical and Translational Research (RCTR) [U54]	U54	Special Emphasis Panel (ZMD1-RN)	No - Health Disparity Populations
Clinical Protocol and Data Management: (Core 011)	NCI	PAR-13-386	Cancer Center Support Grants (CCSGs) for NCI-Designated Cancer Centers (P30)	P30	Subcommittee A - Cancer Centers (NCI-A)	No - Health Disparity Populations
E-Cigarette Understanding and Use Among A Diverse Sample of Ethnic Minority Adolescents	NCI	PAR-14-007	Nci Small Grants Program for Cancer Research (Nci Omnibus R03)	R03	Special Emphasis Panel (ZCA1-TCRB-B (M2))	No
Informing the Adaptation of A CHW Model to Facilitate Lung Cancer Screening For Chinese Taxi Drivers	NCI	PAR-14-007	Nci Small Grants Program for Cancer Research (Nci Omnibus R03)	R03	Special Emphasis Panel (ZCA1-SRB-2 (M1))	No
Project 1 - Ethnic Differences in Smoking-Related Biomarkers and Risk of Lung Cancer	NCI	PAR-15-023	National Cancer Institute Program Project Applications (P01)	P01	Special Emphasis Panel (ZCA1-RPRB-C (M1))	No
University of Guam/Cancer Research Center of Hawaii Partnership (1 of 2)	NCI	PAR-15-103	Comprehensive Partnerships to Advance Cancer Health Equity (CPACHE) (U54)	U54	Special Emphasis Panel (ZCA1-SRLB-D (O2))	Yes-AAs/NHPIs

© 2019 Đoàn LN et al. *JAMA Network Open.*

Mglide RCT: A Clinical Glide Path to Close the Guideline-to-Practice Gap in Htn Management	NHLBI	PAR-15-279	Strategies to Increase Delivery of Guideline-Based Care to Populations with Health Disparities (R01)	R01	Special Emphasis Panel (ZRG1-HDM-G (59))	No - Health Disparity Populations
Acupressure Intervention to Improve Fatigue and Physical Functioning of Chinese Immigrant Breast Cancer Survivors	NCI	PAR-15-340	Nci Exploratory/Developmental Research Grant Program (Nci Omnibus R21)	R21	Special Emphasis Panel (ZCA1-SRB-2 (M1))	No
Epidemiology of Age-Related Dementia, Mild Cognitive Impairment and Brain Pathology in A Multiethnic Cohort of Oldest-Old	NIA	PAR-15-356	Major Opportunities for Research in Epidemiology of Alzheimer's Disease and Cognitive Resilience (R01)	RF1	Special Emphasis Panel (ZRG1-PSE-P (59)R)	No - Health Disparity Populations
CSULB Marc U*Star Training Program	NIGMS	PAR-16-113	Maximizing Access to Research Careers Undergraduate - Student Training in Academic Research (MARC U-STAR) (T34)	T34	Training and Workforce Development Subcommittee - C (TWD-C)	Yes-AAs/NHPIs
Community Outreach and Engagement	NCI	PAR-17-095	Cancer Center Support Grants (CCSGs) for NCI-Designated Cancer Centers (P30)	P30	Subcommittee A - Cancer Centers (NCI-A)	No - Health Disparity Populations
Community Outreach and Engagement	NCI	PAR-17-095	Cancer Center Support Grants (CCSGs) for NCI-Designated Cancer Centers (P30)	P30	Subcommittee A - Cancer Centers (NCI-A)	No - Health Disparity Populations
University of Hawaii Cancer Center CCSG	NCI	PAR-17-095	Cancer Center Support Grants (CCSGs) for NCI-Designated Cancer Centers (P30)	P30	Subcommittee A - Cancer Centers (NCI-A)	No - Health Disparity Populations
Predicting and Reducing Future Health Disparities For U.S. Adults With Diabetes	NIMHD	PAR-18-331	Simulation Modeling and Systems Science to Address Health Disparities (R01Clinical Trial Not Allowed)	R01	Biomedical Computing and Health Informatics Study Section (BCHI)	Yes-AAs/NHPIs
Community Outreach Core	NCI	PAR-18-361	Comprehensive Partnerships to	U54	Special Emphasis Panel	Yes-AAs/

© 2019 Đoàn LN et al. *JAMA Network Open.*

			Advance Cancer Health Equity (CPACHE) (U54 Clinical Trial Optional)		(ZCA1-SRB-2 (A1))	NHPIs
Administrative Core	NCI	PAR-18-361	Comprehensive Partnerships to Advance Cancer Health Equity (CPACHE) (U54 Clinical Trial Optional)	U54	Special Emphasis Panel (ZCA1-SRB-2 (A1))	Yes-AAs/ NHPIs
Outreach Core	NCI	PAR-18-361	Comprehensive Partnerships to Advance Cancer Health Equity (CPACHE) (U54 Clinical Trial Optional)	U54	Special Emphasis Panel (ZCA1-SRB-2 (A1))	Yes-AAs/ NHPIs
Outreach Core	NCI	PAR-18-361	Comprehensive Partnerships to Advance Cancer Health Equity (CPACHE) (U54 Clinical Trial Optional)	U54	Special Emphasis Panel (ZCA1-SRB-2 (A1))	Yes-AAs/ NHPIs
Outreach Core	NCI	PAR-18-361	Comprehensive Partnerships to Advance Cancer Health Equity (CPACHE) (U54 Clinical Trial Optional)	U54	Special Emphasis Panel (ZCA1-SRB-2 (A1))	Yes-AAs/ NHPIs
Recovering Nation--Drug Treatment and Decolonization	NIDA	PAR-93--40		F31	Epidemiology and Prevention Research Subcommittee (NIDA-G)	
HIV Risk Among South Asian Women in the Us	NIMH	PAR-94-002	Behavioral Science Track Award for Rapid Transition	R03	Special Emphasis Panel (SRCM-M)	Yes-AAs/ NHPIs
Trends in Alcohol Misuse Among Minority Adolescents	NIAAA	PAR-95-024	Secondary Analysis of Alcohol Abuse Prevention Research Data	R21	Special Emphasis Panel (ZAA1-CC (M3))	No
Correlates of Affective Distress Among Asian Americans	NIMH	PAR-95-040	Scientist Development Award for New Minority Faculty	K01	Clinical Psychopathology Review Committee (CPP)	Yes-AAs/ NHPIs
Detection of Gastric Metaplasia in Asian Americans	NCI	PAR-95-078	Small Grants for Clinical Trials in Digestive and Nutritional Disorders	R21	Special Emphasis Panel (SRC-C)	No

© 2019 Đoàn LN et al. *JAMA Network Open.*

Mental Health of Japanese-American Older Adults	NIMH	PAR-97-015	Nimh Small Grants Program	R03	Special Emphasis Panel (ZRG1-SNEM-3 (01)S)	No
Korean-American Parenting and Adolescent Adaptation	NINR	PAR-97-016	National Institute of Nursing Research National Research Service Award Individual Predoctoral Fellowships	F31	Special Emphasis Panel (NRRC (15))	No
Predictors of Adolescent Drug Use Among Inner-City Youth	NIDA	PAR-97-038	Nida Small Grants Program	R03	Epidemiology and Prevention Research Subcommittee (NIDA-G)	No
Cross Cultural Families Study	NIDA	PAR-97-046	Behavioral Science Track Awards for Rapid Transition-Nida	R03	Special Emphasis Panel (ZDA1-LXF-L (31))	No
Statistical Analysis of Cancer Incidence	NCI	PAR-98-023	Small Grants Program for Cancer Epidemiology	R03	Special Emphasis Panel (ZCA1-GRB-S (M2))	No
Cancer Screening Behavior in Korean American Women	NCI	PAR-99-006	Small Grants Program for Behavioral Research in Cancer Control	R03	Special Emphasis Panel (ZCA1-SRRB-X (O3))	No
Cultural Beliefs and Breast Cancer Screening	NCI	PAR-99-006	Small Grants Program for Behavioral Research in Cancer Control	R03	Special Emphasis Panel (ZCA1-SRRB-X (J1))	No
Impact of Culture on Cancer Screening in Chinese Women	NCI	PAR-99-108	Cancer Prevention, Control and Population Sciences Career Development Award	K07	Subcommittee G - Education (NCI-G)	No
Migratory Responses to Recent U.S. Immigration	NICHD	PAR-99-126	Nichd Small Grants Program	R03	Special Emphasis Panel (CHHD (DB)-G)	No
Factors of Problem Behaviors Among Ethnic Minority Youth	NIMH	PAR-99-139	Underrepresented Minority Dissertation Research Grants in Mental Health	R03	Special Emphasis Panel (ZMH1-BRB-K (04))	No - Health Disparity Populations
Asian American Adolescents' Health Risk Behaviors	NIMH	PAR-99-139	Underrepresented Minority Dissertation Research Grants in Mental Health	R03	Special Emphasis Panel (ZMH1-BRB-K (01))	No - Health Disparity Populations

© 2019 Đoàn LN et al. *JAMA Network Open.*

Cultural Socialization of Korean Adoptees	NIMH	PAR-99-169	Scientist Development Award for New Minority Faculty	K01	Special Emphasis Panel (ZMH1-BRB-S (03))	No - Health Disparity Populations
Men of ASIa Testing For HIV (Math)	NICHD	PAS-00-136	Demographic Research on Sexual Behaviors Related to HIV	R01	Behavioral and Social Consequences of HIV/AIDS Study Section (BSCH)	No - Health Disparity Populations
Metabolic Syndrome As Women Undergo Menopausal Transition: A Multi-Ethnic Study	NIA	PAS-10-226	Advancing Novel Science in Women's Health Research (ANSWHR) (R21)	R21	Kidney, Nutrition, Obesity and Diabetes Study Section (KNOD)	No
Alcohol Outlet Density, Alcohol Beverages & Homicide	NIAAA	RFA-AA-02-002	Research on Alcohol Health Disparities	R01	Special Emphasis Panel (ZAA1-BB (10))	Yes-AAs/NHPIs
Core--Education and Information Transfer Core	NIA	RFA-AG-04-011	Alzheimer's Disease Research Centers	P50	Special Emphasis Panel (ZAG1-ZIJ-7 (J4))	No - Health Disparity Populations
Core--Education and Information Transfer Core	NIA	RFA-AG-04-011	Alzheimer's Disease Research Centers	P50	Special Emphasis Panel (ZAG1-ZIJ-7 (J4))	No - Health Disparity Populations
Mid-Life Aging & Urinary Incontinence in A Multi-Ethnic	NIA	RFA-AG-05-004	Aging Through the Life Span: Longitudinal Data Analyses	R01	Special Emphasis Panel (ZRG1-HOP-R (50)R)	No
Neuropathology Core	NIA	RFA-AG-05-010	Alzheimer's Disease Core Centers	P30	Special Emphasis Panel (ZAG1-ZIJ-4 (M1))	No - Health Disparity Populations
Anemia and Its Relationship With Sarcopenia, Physical Function, and Mortality	NIA	RFA-AG-06-002	Anemia in the Elderly	R01	Special Emphasis Panel (ZAG1-ZIJ-8 (O1))	No - Health Disparity

© 2019 Đoàn LN et al. *JAMA Network Open.*

						Populations
Education and Outreach Core	NIA	RFA-AG-09-001	Alzheimer’s Disease Research Centers (P50)	P50	Special Emphasis Panel (ZAG1-ZIJ-4 (J1))	No - Health Disparity Populations
Alzheimer Disease Research Center	NIA	RFA-AG-10-002	Alzheimer’s Disease Research Centers (P50)	P50	Special Emphasis Panel (ZAG1-ZIJ-4 (J2))	No - Health Disparity Populations
Core--Education and Information Transfer Core	NIA	RFA-AG-10-002	Alzheimer’s Disease Research Centers (P50)	P50	Special Emphasis Panel (ZAG1-ZIJ-7 (J4))	No - Health Disparity Populations
Core--Education and Information Transfer Core	NIA	RFA-AG-10-002	Alzheimer’s Disease Research Centers (P50)	P50	Special Emphasis Panel (ZAG1-ZIJ-7 (J4))	No - Health Disparity Populations
Investigator Development Core	NIA	RFA-AG-12-012	Resource Centers for Minority Aging Research (RCMAR) and Coordinating Center (P30)	P30	Special Emphasis Panel (ZAG1-ZIJ-3)	No - Health Disparity Populations
Education and Outreach Core	NIA	RFA-AG-13-019	Alzheimer's Disease Research Centers (P50	P50	Special Emphasis Panel (ZAG1-ZIJ-4 (J1))	No - Health Disparity Populations
the Johns Hopkins Alzheimer's Disease Resource Center For Minority Aging Research - Admin Core	NIA	RFA-AG-18-002	Alzheimer's-Related Resource Centers for Minority Aging Research (AD-RCMAR) (P30)	P30	Special Emphasis Panel (ZAG1-ZIJ-9 (J1))	No - Health Disparity Populations
Administrative Core (Anc)	NIA	RFA-AG-18-003	Resource Centers for Minority Aging Research (RCMAR) (P30)	P30	Special Emphasis Panel (ZAG1-ZIJ-9 (J2))	No - Health Dispa

© 2019 Đoàn LN et al. *JAMA Network Open.*

						rity Populations
Investigator Development Core	NIA	RFA-AG-18-003	Resource Centers for Minority Aging Research (RCMAR) (P30)	P30	Special Emphasis Panel (ZAG1-ZIJ-3)	No - Health Disparity Populations
Falls and Fractures Among Elderly Japanese Americans	NIA	RFA-AG-91--03		R01	Special Emphasis Panel (NBSA (70))	
Long Term Care Use in Japanese American Elderly	NIA	RFA-AG-92--02		R01	Special Emphasis Panel (NBSA (32))	
Care-Seeking Strategies in Families of Ethnic Dementia-Affected Elderly	NIA	RFA-AG-93--04		P20	Special Emphasis Panel (ZAG1-CLL-8 (20))	
Effect of Smoking Cessation Interventions in A Chinese *	NCI	RFA-CA-01-017	Research Answers to Nci's Provocative Questions (R01)	R01	Special Emphasis Panel (ZCA1-SRRB-K (01))	No - Health Disparity Populations
Preventive Health Care Use in Elderly Cancer Survivors	NIA	RFA-CA-04-003	Long Term Cancer Survivors: Research Initiatives	R01	Special Emphasis Panel (ZCA1-SRRB-Y (F1))	No - Health Disparity Populations
Racial and Socioeconomic Disparities in Lung Cancer: A Multifactorial Analysis	NCI	RFA-CA-09-001	NIH-Supported Centers for Population Health and Health Disparities (Cphhd) (P50)	P50	Special Emphasis Panel (ZCA1-SRLB-3 (J1))	No - Health Disparity Populations
Pilot Project	NCI	RFA-CA-09-032	Community Networks Program (Cnp) – Centers for Reducing Cancer Disparities Through Outreach, Research and Training (U54)	U54	Special Emphasis Panel (ZCA1-PCRB-G (M1))	Yes-AAs/NHPIs
'Imi Hale Native Hawaiian Cancer Network	NCI	RFA-CA-09-032	Community Networks Program (Cnp) – Centers for Reducing Cancer Disparities Through Outreach, Research and Training (U54)	U54	Special Emphasis Panel (ZCA1-PCRB-G (M1))	Yes-AAs/NHPIs

© 2019 Đoàn LN et al. *JAMA Network Open.*

Aancart: the National Center For Reducing Asian American Cancer Health Disparitie	NCI	RFA-CA-09-032	Community Networks Program (Cnp) – Centers for Reducing Cancer Disparities Through Outreach, Research and Training (U54)	U54	Special Emphasis Panel (ZCA1-PCRB-G (M1))	Yes-AAs/NHPIs
Alameda County Network Program For Reducing Cancer Disparities	NCI	RFA-CA-09-032	Community Networks Program (Cnp) – Centers for Reducing Cancer Disparities Through Outreach, Research and Training (U54)	U54	Special Emphasis Panel (ZCA1-PCRB-G (M1))	Yes-AAs/NHPIs
Asian Community Cancer Health Disparities Center (Acchdc)	NCI	RFA-CA-09-032	Community Networks Program (Cnp) – Centers for Reducing Cancer Disparities Through Outreach, Research and Training (U54)	U54	Special Emphasis Panel (ZCA1-PCRB-G (M1))	Yes-AAs/NHPIs
Development of Breast Cancer Risk Model For the Pacific	NCI	RFA-CA-09-501	Comprehensive Minority Institution/Cancer Center Partnership (Limited Competition U54)	U54	Special Emphasis Panel (ZCA1-SRLB-D (O2))	Yes-AAs/NHPIs
University of Guam/Cancer Research Center of Hawaii Partnership (1 of 2)	NCI	RFA-CA-09-501	Comprehensive Minority Institution/Cancer Center Partnership (Limited Competition U54)	U54	Special Emphasis Panel (ZCA1-SRLB-D (O2))	Yes-AAs/NHPIs
Developmental Research Program	NCI	RFA-CA-17-033	Feasibility and Planning Studies for Development of Specialized Programs of Research Excellence (Spores) to Investigate Cancer Health Disparities (P20)	P20	Special Emphasis Panel (ZCA1-RPRB-N (A1))	Yes-AAs/NHPIs
Breast Cancer Treatment Protocols For Hawaii Minorities	NCI	RFA-CA-93--27		R01	Special Emphasis Panel (SRC (78))	
Breast Cancer in Young Women--Population Based Approach	NCI	RFA-CA-93--33		R01	Special Emphasis Panel (SRC (78))	
Norris Cancer Center Breast Cancer Research Program	NCI	RFA-CA-94--05		R21	Special Emphasis Panel (SRC (05))	
Breast & Cervical Cancer Screening Among Filipino Women	NCI	RFA-CA-96-013	Translational Investigator Grants for Cancer Prevention and Control	R01	Special Emphasis Panel (ZCA1-GRB-H (J1))	No

© 2019 Đoàn LN et al. *JAMA Network Open.*

Well Being in Longterm Multiethnic Prostate Ca Survivors	NIA	RFA-CA-97-018	Long-Term Cancer Survivors: Research Initiatives	R01	Special Emphasis Panel (ZCA1-RLB-X (M2))	No
Periodontal Disease in Diabetic Women With Preterm Birth	NIDCR	RFA-DE-02-004	Pilot Grants for Research to Prevent Or Reduce Oral Health Disparities	R21	Special Emphasis Panel (ZDE1-AS (67))	Yes-AAs/NHPIs
A Study of Oral Health Disparities in Adult Asian & Pac*	NIDCR	RFA-DE-02-005	Planning Grants for Research to Prevent Or Reduce Oral Health Disparities	R21	Special Emphasis Panel (ZDE1-AS (67))	Yes-AAs/NHPIs
Bayanihan (Working Together to Help): A Filipino Educat*	NIDDK	RFA-DK-02-019	Minority Organ and Tissue Donation	R01	Special Emphasis Panel (ZDK1-GRB-9 (M2)S)	No - Health Disparity Populations
Multimedia Intervention to Motivate Ethnic Teens to Be Designated Donors	NIDDK	RFA-DK-06-016	Minority Organ and Tissue Donation (R01)	R01	Special Emphasis Panel (ZDK1-GRB-R (M1)S)	No - Health Disparity Populations
Core to Support Underserved Or Health Disparity Populations	NIDDK	RFA-DK-15-003	NIDDK Centers for Diabetes Translation Research (P30)	P30	Special Emphasis Panel (ZDK1-GRB-1 (M2)P)	No - Health Disparity Populations
Understanding and Planning Donation Information Needs of Asian American Communities	NIDDK	RFA-DK-16-022	Promoting Organ and Tissue Donation Among Diverse Populations (R01)	R01	Special Emphasis Panel (ZDK1-GRB-J (M1)S)	Yes-AAs/NHPIs
Increasing Minority Population Awareness Through Community Teaching For Improved Organ Donation (Impact For Improved Organ Donation)	NIDDK	RFA-DK-16-022	Promoting Organ and Tissue Donation Among Diverse Populations (R01)	R01	Special Emphasis Panel (ZDK1-GRB-J (M1)S)	Yes-AAs/NHPIs
Cultural-Based Diabetes Program For Native Hawaiians	NIDDK	RFA-DK-93--23		R01	Special Emphasis Panel (DDK (23))	
Reduction of Diabetic Risk in Japanese Americans	NIDDK	RFA-DK-93--23		R01	Special Emphasis Panel (DDK (23))	
Effects of Volcanic Air Pollution on Respiratory Health	NIEHS	RFA-ES-01-003	Community-Based Participatory Research in Environmental Health	R01	Special Emphasis Panel (ZES1-JPM-B (CB))	No - Health Dispa

© 2019 Đoàn LN et al. *JAMA Network Open.*

						rity Populations
Community Outreach and Education Core	NIEHS	RFA-ES-11-001	Environmental Health Sciences Core Center Grants (P30)	P30	Special Emphasis Panel (EHS (P3))	No
Community Engagement Core	NIEHS	RFA-ES-16-001	Environmental Health Sciences Core Centers (Ehs Cc) (P30)	P30	Special Emphasis Panel (ZES1-LWJ-J (P3)1)	No
Asian Immigrant Community Institutions in Nyc and HIV	NICHD	RFA-HD-01-017	Developmental Mechanisms of Human Structural Birth Defects (P01)	R21	Special Emphasis Panel (ZHD1-DSR-W (17))	No
Chinese American Fathers With Children With Mr/Dd	NICHD	RFA-HD-93--14		R01	Special Emphasis Panel (SRC (14))	
Does Shared Decision-Making Improve Adherence in Asthma?	NHLBI	RFA-HL-01-005	Overcoming Barriers to Treatment Adherence in Minorities and Persons Living in Poverty	R01	Special Emphasis Panel (ZRG1-RPHB-4 (02)R)	Yes-AAs/NHPIs
Chinese Community Smoking Cessation Project	NHLBI	RFA-HL-01-005	Overcoming Barriers to Treatment Adherence in Minorities and Persons Living in Poverty	R01	Special Emphasis Panel (ZRG1-RPHB-4 (02)R)	Yes-AAs/NHPIs
New Strategies to Enhance Drug Adherence in Hypertension	NHLBI	RFA-HL-01-005	Overcoming Barriers to Treatment Adherence in Minorities and Persons Living in Poverty	R01	Special Emphasis Panel (ZRG1-RPHB-4 (02)R)	Yes-AAs/NHPIs
Partnerships For Diabetes Related Disparties in Hawaii	NIMHD	RFA-MD-02-002		P20	Special Emphasis Panel (ZMD1-TC (02))	
Partnerships For Diabetes Related Disparities in Hawaii	NIMHD	RFA-MD-02-002		P20	Special Emphasis Panel (ZMD1-TC (02))	
Research Education and Training Core	NIMHD	RFA-MD-05-003	Request for Applications NCMHD Centers of Excellence in Partnerships for Community Outreach, Research on Health Disparities and Training (Project EXPORT – Establishing Exploratory Centers)	P20	Special Emphasis Panel (ZMD1)	No - Health Disparity Populations

© 2019 Đoàn LN et al. *JAMA Network Open.*

Health Disparities Research At Sirc: Cultural Processes in Risk and Resiliency	NIMHD	RFA-MD-06-003	Establishing Exploratory NCMHD Research Centers of Excellence (P20)	P20	Special Emphasis Panel (ZRG1-DIG-C (52)R)	Yes-AAs/NHPIs
Partnerships For Diabetes Related Disparities in Hawaii	NIMHD	RFA-MD-06-003	Establishing Exploratory NCMHD Research Centers of Excellence (P20)	P20	Special Emphasis Panel (ZMD1-TC (02))	Yes-AAs/NHPIs
Faith-Based Partnership Promoting Korean Parenting and Child Mental Health	NIMHD	RFA-MD-10-004	NCMHD Innovative Faith-Based Approaches to Health Disparities Research (R21)	R21	Special Emphasis Panel (ZMD1-PA (07)S)	Yes-AAs/NHPIs
Faith in Action Research Alliance: Reducing Diabetes Among Pacific Islanders	NIMHD	RFA-MD-10-004	NCMHD Innovative Faith-Based Approaches to Health Disparities Research (R21)	R21	Special Emphasis Panel (ZMD1-PA (07)S)	Yes-AAs/NHPIs
Innovative Faith-Based Education on Advance Directives in Asian American Communit	NIMHD	RFA-MD-10-004	NCMHD Innovative Faith-Based Approaches to Health Disparities Research (R21)	R21	Special Emphasis Panel (ZMD1-PA (07)S)	Yes-AAs/NHPIs
Partnerships For Diabetes Related Disparities in Hawaii	NIMHD	RFA-MD-11-002	NIMHD Exploratory Centers of Excellence (P20)	P20	Special Emphasis Panel (ZMD1-TC (02))	Yes-AAs/NHPIs
Filial Piety and Psychosocial Well-Being in Chinese Population	NIMHD	RFA-MD-12-001	Nimhd Health Disparities Research (R01)	R01	Special Emphasis Panel (ZMD1-MLS (01))	Yes-AAs/NHPIs
Disparities in Chronic Illness Care For Patients With Language Barriers	NIMHD	RFA-MD-12-001	Nimhd Health Disparities Research (R01)	R01	Special Emphasis Panel (ZMD1-MLS (01))	Yes-AAs/NHPIs
Causes: Causes of Asian American Mortality Understood By Socio-Economic Status	NIMHD	RFA-MD-12-001	Nimhd Health Disparities Research (R01)	R01	Special Emphasis Panel (ZMD1-MLS (01))	Yes-AAs/NHPIs
Epidemiology of Suicidal Behavior in Racially/Ethnically Diverse Older Americans	NIMHD	RFA-MD-12-001	Nimhd Health Disparities Research (R01)	R01	Special Emphasis Panel (ZMD1-MLS (01))	Yes-AAs/NHPIs
Substance Abuse & Treatment Gaps in Asians, Pacific Islanders & Multiple-Race Ind	NIMHD	RFA-MD-12-003	NIMHD Transdisciplinary Collaborative Centers for Health Disparities Research [U54]	R01	Special Emphasis Panel (ZMD1-MLS (01))	No - Health Disparity Populations
Collaborations and Partnerships Core	NIMHD	RFA-MD-13-003	NIMHD Transdisciplinary Collaborative Centers for Health Disparities Research [U54]	U54	Special Emphasis Panel (ZMD1-RN)	No - Health Disparity

						Populations
Disparities in Acute Psychiatric Care: System, Community & Economic Determinants	NIMHD	RFA-MD-13-006	NIMHD Social, Behavioral, Health Services, and Policy Research on Minority Health and Health Disparities (R01)	R01	Special Emphasis Panel (ZMD1-DRI (01))	Yes-AAs/NHPIs
Insurance Instability and Disparities in Chronic Disease Outcomes	NIMHD	RFA-MD-13-006	NIMHD Social, Behavioral, Health Services, and Policy Research on Minority Health and Health Disparities (R01)	R01	Special Emphasis Panel (ZMD1-DRI (01))	Yes-AAs/NHPIs
Advancing Health Equity Through Multi-Level Cultural Determinants Research	NIMHD	RFA-MD-14-003	Limited Competition: NIMHD Exploratory Centers of Excellence Pilot Research Projects (P20)	P20	Special Emphasis Panel (ZMD1-MLS (04))	No - Health Disparity Populations
Engaging NHPIs and Activating Communities to Take Steps (Enacts)	NIMHD	RFA-MD-15-014	NIMHD Transdisciplinary Collaborative Centers for Health Disparities Research on Chronic Disease Prevention (U54)	U54	Special Emphasis Panel (ZMD1-MLS (12))	Yes-AAs/NHPIs
Building Strengths and Inspiring Hope Among Youth and Their Communities	NIMHD	RFA-MD-17-003	Research Centers in Minority Institutions (RCMI) (U54)	U54	Special Emphasis Panel (ZMD1-MLS (O1))	Yes-AAs/NHPIs
Research Infrastructure Core	NIMHD	RFA-MD-17-003	Research Centers in Minority Institutions (RCMI) (U54)	U54	Special Emphasis Panel (ZMD1-MLS (O1))	Yes-AAs/NHPIs
Nyu Center For the Study of Asian American Health (Csaah)	NIMHD	RFA-MD-17-005	NIMHD Specialized Centers of Excellence on Minority Health and Health Disparities (U54)	U54	Special Emphasis Panel (ZMD1-XLN (O2))	Yes-AAs/NHPIs
Unpacking the Mechanisms of Disparities For HIV-Related Hypertension in African American and Asian Pacific American Msm	NIMHD	RFA-MD-18-002	Mechanisms of Disparities for HIV-Related Co-Morbidities in Health Disparity Populations (R01-Clinical Trial Not Allowed)	R01	Special Emphasis Panel (ZMD1-XLN (O1))	Yes-AAs/NHPIs
Developing Ptsd Risk Assessment Tools Using the World Trade Center Outcome Study	NIMH	RFA-MH-09-060	Network(S) for Developing PTSD Risk Assessment Tools (R21)	R21	Special Emphasis Panel (ZMH1-ERB-N (04))	No
Epidemiology of Torture and Trauma in Two Refugee Groups	NIMH	RFA-MH-98-012	Mental Health Research for	R01	Special Emphasis Panel	No - Health

© 2019 Đoàn LN et al. *JAMA Network Open.*

			Survivors of Torture and Related Trauma		(ZMH1-BRB-S (05))	Dispa rity Popul ations
Breast and Cervical Cancer Screening in Korean-Americans	NIN R	RFA-NR-01-002	NINR Mentored Research Scientist Development Award for Minority Investigators	K01	Special Emphasis Panel (ZNR1-REV-A (31))	No - Healt h Dispa rity Popul ations
Korean American Parent Training	NIN R	RFA-NR-02-002	NINR Mentored Research Scientist Development Award for Minority Investigators	K01	Special Emphasis Panel (ZNR1-REV-A (45))	No - Healt h Dispa rity Popul ations
Emotional Distress and Risky Behaviors in Asian Youth	NIN R	RFA-NR-02-002	NINR Mentored Research Scientist Development Award for Minority Investigators	K01	Special Emphasis Panel (ZNR1-REV-A (45))	No - Healt h Dispa rity Popul ations
Center For 'Ohana Self-Management of Chronic Illnesses Hawaii (Cosmci0): Building	NIN R	RFA-NR-07-004	Nursing Science Centers in Self-Management Or End-of-Life Research: Building Research Teams for the Future (P20)	P20	Special Emphasis Panel (ZNR1-REV-B (04)R)	No - Healt h Dispa rity Popul ations
Los Angeles Stroke Prevention/Intervention Research Program in Health Disparities	NIN DS	RFA-NS-12-007	Stroke Prevention/Interventio n Research Program (Spirp) (U54)	U54	Special Emphasis Panel (ZNS1-SRB-N (02))	Yes-AAs/ NHPI s
Partners in Research Program With Chinese Community	NIA	RFA-OD-07-001	NIH Partners in Research Program (R03)	R03	Special Emphasis Panel (ZHD1-DSR-H (OD))	No
Partners in Research Program With Chinese Community	NIA	RFA-OD-07-001	NIH Partners in Research Program (R03)	R03	Special Emphasis Panel (ZHD1-DSR-H (OD))	No
Maximizing Immigrant and Refugee Health: A Community-Partnered Approach	NID A	RFA-OD-07-001	NIH Partners in Research Program (R03)	R03	Special Emphasis Panel (ZHD1-DSR-H (OD))	No
Unjust Targeting: How Marketing Features Impact Consumer Response and Tobacco Use	NID A	RFA-OD-13-014	Mentored Research Scientist Career Development Award in Tobacco Control Regulatory Research (K01)	K01	Special Emphasis Panel (ZRG1-BST-N (50)R)	No - Healt h Dispa rity Popul ations

Project 2	NCI	RFA-OD-17-006	Tobacco Centers of Regulatory Science for Research Relevant to the Family Smoking Prevention and Tobacco Control Act (U54)	U54	Special Emphasis Panel (ZRG1-IFCN-L (40))	No - Health Disparity Populations
Transforming Cancer Knowledge, Attitudes and Behavior Through Narrative	NCI	RFA-RM-08-029	Roadmap Transformative R01 Program (R01)	R01	Special Emphasis Panel (ZRG1-BCMB-A (51)R)	No
Clinical Trial: Ccre-Curedm	NCRR	RFA-RR-05-001	Technology Development for Biomedical Applications	P20	Special Emphasis Panel (RIRG (01)-M)	No
Bnp Heart Function	NCRR	RFA-RR-99-005	Centers of Clinical Research Excellence At RCMI Eligible Institutions with Medical Schools	U54	Special Emphasis Panel (ZRR1-RI-1 (01))	No - Health Disparity Populations
the Center of Urban Research and Education in Diabetes	NCRR	RFA-RR-99-005	Centers of Clinical Research Excellence At RCMI Eligible Institutions with Medical Schools	U54	Special Emphasis Panel (ZRR1-RCMI-1 (01))	No - Health Disparity Populations

FOA Funding opportunity Announcement, *IC* Institute/Center, *NCCAM* National Center for Complementary and Integrative Health, *NCI* National Cancer Institute, *NCMHD* (transitioned into the National Institute of Minority Health and Health Disparities [NIMHD] in 2010) National Center on Minority Health and Health Disparities, *NCRR* National Center for Research Resources, *NHLBI* National Heart, Lung, and Blood Institute, *NIA* National Institute on Aging, *NIAAA* National Institute on Alcohol Abuse and Alcoholism, *NIDA* National Institute on Drug Abuse, *NIDCD* National Institute on Deafness and Other Communication Disorders, *NIDCHD* Eunice Kennedy Shriver National Institute of Child Health and Human Development, *NIDDK* National Institute of Diabetes and Digestive and Kidney Diseases, *NIEHS* National Institute of Environmental Health Sciences, *NIGMS* National Institute of General Medical Sciences, *NIMH* National Institute of Mental Health, *NIMHD* National Institute of Minority Health and Health Disparities, *NINR* National Institute of Nursing Research

[a] Includes all projects by unique FOA. Some projects were funded by more than one FOA.

[b] Administering IC is listed. the administering IC is the agency responsible for overseeing the research grant or the agency funding the research or both.

[c] The types of FOAs includes program announcements (PA), a program announcement with special receipt, referral and/or review considerations (PAR), and request for applications (RFA). the format of FOAs is written as the type of solicitation, administering IC, fiscal year of the solicitation, and associated serial number.

[d] The group responsible for the review of grant applications in a particular scientific area.

eTable2. Proportions of Total NIH Expenditures by AA/NHPI Grouping Category, federal fiscal year 1992 - 2018

	Total NIH	AA/NHPI Only		Total AA/NHPI	
	Funding, $	Funding, $	% of Total NIH Funding	Funding, $	% of Total NIH Funding
1992	7,260,565,000	535,936	0.01%	535,936	0.01%
1993	7,459,476,000	-	-	-	-
1994	7,720,806,000	224,584	0.00%	224,584	0.00%
1995	7,912,237,000	4,550,299	0.06%	9,549,405	0.12%
1996	8,413,053,000	4,372,171	0.05%	4,576,309	0.05%
1997	8,992,772,000	10,279,643	0.11%	18,790,941	0.21%
1998	9,738,629,000	8,562,783	0.09%	18,380,384	0.19%
1999	11,162,176,000	9,250,688	0.08%	22,349,966	0.20%
2000	12,856,165,000	8,973,222	0.07%	23,649,128	0.18%
2001	14,867,387,000	12,491,173	0.08%	24,902,178	0.17%
2002	16,790,120,000	14,161,258	0.08%	29,819,937	0.18%
2003	18,502,396,000	13,088,255	0.07%	29,040,008	0.16%
2004	19,565,958,000	15,976,341	0.08%	37,831,135	0.19%
2005	19,923,201,000	13,658,754	0.07%	34,610,625	0.17%
2006	20,007,151,000	17,017,837	0.09%	35,952,821	0.18%
2007	20,322,309,000	17,823,836	0.09%	38,560,192	0.19%
2008	20,623,023,000	19,858,087	0.10%	36,714,635	0.18%
2009	21,119,161,000	23,293,625	0.11%	40,167,756	0.19%
2010	21,574,246,000	28,789,966	0.13%	48,034,530	0.22%
2011	21,437,829,000	25,020,814	0.12%	43,812,981	0.20%
2012	21,563,359,000	30,233,713	0.14%	47,739,497	0.22%
2013	20,032,695,000	23,314,785	0.12%	36,350,929	0.18%
2014	20,781,240,000	18,701,211	0.09%	36,777,145	0.18%
2015	20,947,134,000	17,755,466	0.08%	29,051,252	0.14%
2016	22,426,509,000	18,826,457	0.08%	34,970,409	0.16%
2017	23,715,131,000	24,848,858	0.10%	40,233,724	0.17%
2018	25,569,347,000	27,687,405	0.11%	52,909,714	0.21%

© 2019 Đoàn LN et al. *JAMA Network Open.*

eTable 3. Estimates of Dollar Amounts Over Time for NIH-Funded Asian American, Native Hawaiian, & Pacific Islander Clinical Research, Federal Fiscal Year 1992-2018

	AA/NHPI Only		AA/NHPI and non-AA/NHPI		Total AA/NHPI	
	Funding (95% CI), $ (thousands)	*P* value	Funding (95% CI), $ (thousands)	*P* value	Funding (95% CI), $ (thousands)	*P* value
Year	15,944 (10,461, 21,427)	**<.001**	9,415 (4,669, 14,160)	**<.001**	12,860 (9,215, 16,504)	**<.001**
Before 2000 (reference)	-	-	-	-	-	-
After 2000	92,962 (13,756, 172,167)	**.02**	-115,265 (-183,009, -47,521)	**<.001**	-6,871 (-59,490, 45,758)	.80

© 2019 Đoàn LN et al. *JAMA Network Open.*

eTable 4. Proportions of Total NIH Clinical Research Expenditures by AA/NHPI Grouping Category, federal fiscal year 2008 - 2018

Year	Total Clinical Research	AA/NHPI Only		Total AA/NHPI	
	Funding, $	Funding, $	% of Clinical Research	Funding, $	% of Clinical Research
2008	9,629,000,000	19,858,087	0.21%	36,714,635	0.38%
2009	10,336,000,000	23,293,625	0.23%	40,167,756	0.39%
2010	10,720,000,000	28,789,966	0.27%	48,034,530	0.45%
2011	10,503,000,000	25,020,814	0.24%	43,812,981	0.42%
2012	10,951,000,000	30,233,713	0.28%	47,739,497	0.44%
2013	10,604,000,000	23,314,785	0.22%	36,350,929	0.34%
2014	11,087,000,000	18,701,211	0.17%	36,777,145	0.33%
2015	11,366,000,000	17,755,466	0.16%	29,051,252	0.26%
2016	12,176,000,000	18,826,457	0.15%	34,970,409	0.29%
2017	12,695,000,000	24,848,858	0.20%	40,233,724	0.32%
2018	13,720,000,000	27,687,405	0.20%	52,909,714	0.38%

© 2019 Đoàn LN et al. *JAMA Network Open.*

eTable 5. Estimates of New Projects Awarded Over Time for NIH-Funded Asian American, Native Hawaiian, & Pacific Islander Clinical Research, Federal Fiscal Year 1992-2018

	AA/NHPI Only		AA/NHPI and non-AA/NHPI		Total AA/NHPI	
	No. of Projects	*P* value	No. of Projects	*P* value	No. of Projects	*P* value
Year	0.21 (-0.05, 0.47)	0.11	0.23 (0.09, 0.55)	0.15	0.44 (-0.03, 0.90)	0.06
Before 2000 (reference)	-	-	-	-	-	-
After 2000	4.72 (0.65, 8.79)	**0.02**	5.17 (0.06, 10.27)	**0.05**	9.89 (2.77, 17.01)	**.01**

© 2019 Đoàn LN et al. *JAMA Network Open.*
